# The effectiveness of psychosocial interventions for reducing problematic substance use, mental ill health, and housing instability in people experiencing homelessness in high income countries: A systematic review and meta‐analysis

**DOI:** 10.1002/cl2.70019

**Published:** 2025-01-17

**Authors:** Chris O'Leary, Esther Coren, Sandor Gellen, Anton Roberts, Harry Armitage

**Affiliations:** ^1^ Department of History, Politics and Philosophy Manchester Metropolitan University Manchester UK; ^2^ Independent Consultant; ^3^ Policy Evaluation and Research Unit Manchester Metropolitan University Manchester UK

**Keywords:** homelessness, psychosocial interventions, systematic review, effectiveness

## Abstract

**Background:**

Adults experiencing homelessness in high income countries often also face issues of problematic substance use, mental ill health, in addition to housing instability, so it is important to understand what interventions might help address these issues. While there is growing evidence of the effectiveness of psychosocial interventions for the general population, limited evidence exists specifically for those experiencing homelessness.

**Objectives:**

To summarise the existing evidence of whether psychosocial interventions work in reducing problematic substance use, mental ill health, and housing instability for adults experiencing homelessness in high income countries.

**Search Methods:**

We used searches undertaken for the Homelessness Effectiveness Evidence and Gap Map (EGM) 5th edition. These were supplemented with hand searches of key journals and a call for evidence.

**Selection Criteria:**

We included all Randomised Control Trials and non‐randomised studies where a comparison group was used and which examined psychosocial interventiONS for adults experiencing homelessness. ‘Psychosocial intervention’ is a broad term and covers several interventions, including cognitive behavioural therapy (CBT), contingency management, and motivational interviewing. We focused on studies that measure at least one of three outcomes: reduction in problematic substance use (alcohol and/or drugs); reduction in mental ill‐health; reduction in housing instability.

**Data Collection and Analysis:**

For included studies sourced from the EGM, we used the risk of bias assessments reported in the EGM. For included studies sourced from our own searches, we used the same tools used in the EGM to undertake our own assessments. We carried out meta‐analysis where possible, and where not possible, presented included studies narratively.

**Findings:**

We included 26 papers covering 23 individual intervention studies. All of the included studies were from the United States. Of the 26 papers, 14 were assessed as having medium or high risk of bias, with main issues being lack of masking/blinding, lack of power calculations, and high levels of drop‐out.

**Effectiveness of Psychosocial Interventions:**

We found that psychosocial interventions overall were better than standard care (−0.25 SD, 95% confidence intervals [CI] [−0.36, −0.13]). This finding covered six different interventions and was subject to a high level of between‐study differences (heterogeneity). We also found that psychosocial interventions were more effective than standard care in relation to all three of our outcomes of interest, although were statistically significant only for substance abuse and mental ill‐health. For substance use, we found an average effect size of (−0.34 SD, 95% CI [−0.48, −0.21]); for mental ill health of (−0.18 SD, 95% CI [−0.34, −0.01]); and for housing instability of (−0.10 SD, 95% [−0.90, 0.70]).

**Effectiveness of Individual Psychosocial Interventions:**

We were able to undertake five meta‐analyses (statistical summaries) with respect to four types of intervention: CBT, Contingency Management, Motivational Interviewing, and Brief Motivational Interventions, in relation to specific outcomes. Of these five analyses, we found significant effects for the effectiveness of Contingency Management in reducing problematic substance use (−0.49 SD, 95% CI [−0.85, −0.14]), and of Motivational Interviewing in reducing mental ill‐health (−0.19 SD, 95% CI [−0.26, −0.12]). We also found non‐significant effects in relation to CBT and reducing mental ill health (−0.30 SD, 95% CI [−0.61, 0.002]), Motivational Interviewing and reducing problematic substance use (−0.27 SD, 95% CI [−0.56, 0.01]), and Brief Motivational Interventions and reducing problematic substance use (−0.24 SD, 95% CI [−0.61, 0.13]). Meta‐analysis was not possible for any other interventions or outcomes.

**Author Conclusions:**

This systematic review sought to understand the effectiveness of psychosocial interventions for adults in high income countries experiencing homelessness, for reducing problematic substance use, reducing mental ill‐health, and increasing housing stability. The review shows potential benefits of these interventions, with some encouraging results for some interventions and outcomes. Where we could calculate effect sizes, these were often small and, in many cases, crossed the line of no effect (i.e., there is a chance that they are equally or less effective than treatment as usual). Significant heterogeneity between studies and high rates of drop‐out in many studies reduces the confidence in the interventions.

There are some limitations with the evidence base. The included studies were entirely from the United States. There was a clear gender bias in the included studies, with nearly two‐thirds of participants being men. (This is despite 4 of the 26 included studies focusing on women only.) We also found that the theoretical basis for the approach of interventions was not sufficiently considered, so it was difficult to understand why the intervention expected the outcomes they measured. Finally, many of the studies included were assessed as having high or medium risk of bias.

## 1 PLAIN LANGUAGE SUMMARY

Psychosocial interventions can be helpful for adults experiencing homelessness.

Many people experiencing homelessness also face issues with problematic substance use and mental ill health, in addition to housing instability. Psychosocial interventions are a group of different approaches that are often used to reduce these issues. This review includes some evidence of whether these interventions work, as well as practice and policy recommendations. It also highlights the need for research to be conducted outside of the United States.

### 1.1 The review in brief

Psychosocial interventions work to reduce problematic substance use and mental ill health for adults experiencing homelessness. There is currently insufficient evidence to confirm that they are effective in decreasing housing instability. Individual psychosocial interventions can also be effective: cognitive behavioural therapy (CBT) reduces problematic substance use and motivational interviewing reduces mental ill health. It is unclear whether other psychosocial interventions work or not.

### 1.2 What is this review about?

Homelessness is often traumatic, with devastating cONSequences for those experiencing it. People experiencing homelessness face significant barriers such as stigma and discrimination when they try to access services, and often ‘fall through the cracks’ between different services. Homelessness is often associated with problematic substance use, mental ill health, offending behaviour, and being victims of crime, It is important to understand which interventions work best to reduce these issues and improve the lives of people experiencing homelessness. Psychosocial interventions are increasingly used to address some of these issues for people experiencing homelessness. This is a broad group of different interventions, which use psychological approaches to enable positive changes in thoughts, motivations, and behaviours. This review looked at whether these interventions worked to reduce problematic substance use (alcohol and/or drugs), mental ill health, and housing instability.

### 1.3 What is the aim of this review

This Campbell systematic review examines existing evidence of the effects of psychosocial interventions on at least one of three outcomes (reducing problematic substance use, mental ill health, and/or housing instability). The review compares psychosocial interventions and other ways of delivering services. The review summarises results from 26 papers, all of which cover studies that were conducted in the United States and almost all of which were randomised controlled trials (RCTs). The authors of this review did not conduct any of the studies presented in the 26 included papers.

### 1.4 What are the main findings of this review?

We examined 898 individual papers to see if they evaluated psychosocial interventions, used with adults experiencing homelessness, in relation to at least one of the three outcomes of interest to us. From these 898 papers, we included 26 papers that covered 23 studies that met our criteria. This process was undertaken by two researchers independently, with a third researcher making final decisions. All of the included papers were from the United States and all but one were RCTs (a type of evaluation where people are randomly assigned to receive either the intervention of interest or a comparison service). The papers covered a number of different psychosocial interventions. We were able to find evidence about six interventions, including Brief Interventions, Brief Motivational Interventions, CBT, Contingency Management, Dialectical Behavioural Therapy, and Motivational Interviewing. We were not able to find evidence about other types of psychosocial interventions.

#### 1.4.1 Do psychosocial interventions work for adults experiencing homelessness?

Yes, overall, psychosocial interventions do work better when compared to other services for adults experiencing homelessness.

Psychosocial interventions work overall in reducing problematic substance use when compared to other services. They also work in reducing mental ill health when compared to other services. But the evidence is less than clear about whether they work in reducing housing instability. This might be because there were only two studies, presented in three papers and all by the same lead author, that looked at the effect of these interventions on housing outcomes.

#### 1.4.2 Which individual psychosocial interventions work, and for what outcomes?

Contingency Management works better than other services in reducing problematic substance use for adults experiencing homelessness. Motivational Interviewing works better than other services in reducing mental ill health for adults experiencing homelessness.

Other results were less encouraging, as it is unclear whether the interventions work for other outcomes. There were non‐significant effects (i.e., they could work better than other services, but there is also a chance that they work equally or less well) in relation to CBT and reducing mental ill health, motivational interviewing and reducing problematic substance use, and brief motivational interventions and reducing problematic substance use. There were no results about whether these six interventions reduced housing instability. There were no results about other types of psychosocial interventions on any of the three outcomes of interest to us.

### 1.5 What do the findings of this review mean?

Psychosocial interventions can help adults experiencing homelessness reduce problematic substance use and reduce mental ill health. Individual interventions including CBT and Motivational Interviewing can also work. But more research is needed about psychosocial interventions, particularly from outside of the United States.

### 1.6 How up‐to‐date is this review?

This review includes papers and studies published before August 2022.

## 2 BACKGROUND

### 2.1 The problem, condition, or issue

#### 2.1.1 The significant and increasing scale of homelessness

Homelessness is a major social and public health concern (MacKnee & Mervyn, 2002; Wright, 2017). In recent years, rates of homelessness are reported to have increased in many high income countries, although differences in definitions and measures mean that it is challenging to get an accurate overall picture (Sullivan, 2023). For example, in the United States, the Department for Housing and Urban Development reported that 582,500 people were experiencing homelessness in early 2022 (deSousa et al., 2022). This is of a similar level to that set out in the State of Homelessness in America report that stated that in January 2020 over 580,000 were experiencing homelessness, and that rates of homelessness had grown by 2% over the previous year (National Alliance To End Homelessness, 2021). In Canada, the most recent estimate suggests that around 35,000 people are experienced homelessness on a single night, with between 250,000 and 300,000 experiencing homelessness a year (Gaetz et al., 2016), as cited in Wong et al. (2020) with some 700,000 people experiencing homelessness in the EU in 2019 (FEANTSA, 2022).

In England, all forms of homelessness rose between 2008 and 2017 (O'Leary & Simcock, 2020). Recent published data suggests that the number of people experiencing street homelessness and who are sleeping rough (unsheltered) in England fell between 2017 and 2021 (snapshot count taken on a single night in Autumn), with a significant fall recorded in 2020. The large drop in 2020 is probably due to accounted for by government responses to the Covid 19 (DLUHC, 2022), though the reasons for reductiONS in 2017, 2018 and 2019 are not yet known. However, recent data suggests an increase between 2021 and 2022, with 3069 people estimated to be sleeping rough on a single night in Autumn 2022 (DLUHC, 2023). In the United Kingdom, the proportion of people experiencing homelessness who are sleeping rough is relatively small compared to other forms of homelessness, with around 280,000 households assessed as either being threatened with homelessness or already homeless in 2021–22 (DLUHC, 2023).

We recognise that homelessness is a complex and multifaceted concept, with differences in how homelessness is understood and experienced, and how these differences are conceptualised and described. During the scoping work for this review, a workshop was held with five people with lived experience to consider the research objectives and definitions used. This workshop developed a new definition of homelessness, building on the previous work of Keenan et al. (2020). This definition is set out in Section 4.

There are also ongoing policy and practice debates around the causes of homelessness, and around interventions aimed at preventing and reducing homelessness. In terms of the causes of homelessness, Glen Bramley and Suzanne Fitzpatrick state that there is significant debate between a focus on individual‐level risks or causes, and structural or systemic causes (such as labour market conditions, housing supply, and poverty). These foci vary between countries and over time, though increasingly it is recognised that both might have explanatory power (Bramley & Fitzpatrick, 2018). These debates often influence policy discussions around the types of interventions that might address homelessness, and whether these should be focused on structural interventions such as increasing housing supply or reducing poverty, or preventing/addressing homelessness at the level of the individual. While individual experiences are highly likely impacted by the structural contexts in which they arise, this review is focused on individual‐level interventions.

Homelessness is almost always a traumatic experience, which can have a devastating effect on those experiencing it (O'Leary et al., 2022). Several studies, some of which are cited below, have highlighted that more visible and extreme forms of homelessness are often associated with adverse childhood events (Koh & Montgomery, 2021), extreme social disadvantage (Mabhala et al., 2017), physical, emotional and sexual abuse (Green et al., 2012; Henny et al., 2007), neglect (Mar et al., 2014), low self‐esteem (Ravikumar et al., 2021), poor physical and mental health (Vallesi et al., 2021), and much lower life expectancy compared to the general population (ONS, 2019). Adults experiencing homelessness (particularly those experiencing the more extreme and visible forms of homelessness, such as rough sleeping/being unsheltered) face significant barriers accessing services, and often fall through the cracks between different services they need to access (Dobson, 2019). They often have repeated, but intermittent, contact with a range of publicly funded services, particularly health (Aldridge et al., 2018), criminal justice (Bramley & Fitzpatrick, 2018), and local government (Dobson, 2019). For example, this population is five times more likely to attend Accident and Emergency (Emergency Room), and three times more likely to be admitted to a hospital, than their housed peers (Cornes, Aldridge, et al., 2018). The existing evidence of effectiveness of interventions for this population is mixed (Luchenski et al., 2018), and there is no specific systematic review on the effectiveness of psychosocial interventions for people experiencing homelessness. Most of the extant evidence base that examines the effectiveness of interventions around homelessness is focused on individual‐level interventions. They are typically aimed at addressing the harms caused by homelessness or reducing homelessness, rather than prevention (O'Leary et al., 2022).

### 2.2 The intervention

#### 2.2.1 Defining psychosocial interventions

There is a lack of a single, agreed definition of psychosocial interventions (Hodges et al., 2011). Many definitions are broad in scope, so that almost any intervention or service might be cONSidered to be ‘psychosocial’ (O'Leary et al., 2022). For example, psychosocial interventions are often defined as being simply non‐pharmacological interventions, which is unhelpful in understanding specifically what counts as a psychosocial intervention (McDermott et al., 2019). Other definitions have some underlying commonalities. These include that psychosocial interventions have a change objective/aim, and that this intended change is psychological, and is often (though not exclusively) focused on mental ill health or problematic substance use. Several definitions include social change as well as psychological change as an objective, and also all exclude interventions that are wholly or mostly pharmacological in approach. But the extant literature also identifies huge variations in these interventions, including differences in setting, intensity, whether the intervention is group or individual based, and the treatment goals of the intervention.

For this review, we used a definition provided by (England et al., 2015) in their report *Psychosocial interventions for mental and substance use disorders: a framework for establishing evidence‐based standards*. They state that psychosocial interventiONS are ‘interpersonal or informational activities, techniques, or strategies that target biological, behavioral, cognitive, emotional, interpersonal, social, or environmental factors’ which aim to make positive changes to the lives of individuals engaging in these activities. This definition is relatively broad. We added further to this definition, to focus on psychosocial interventions that are: (a) formally (though not necessarily universally) recognised as being psychosocial interventions; (b) are structured or planned, with an explicit intended goal or objective; (c) excludes pharmacological interventions (or interventions that are predominately pharmacological in nature); and (d) targeted for use with adults experiencing homelessness. Our focus here is on psychosocial interventions that target individuals. Given this focus, we identified a list of 20 interventions that are the primary focus of this review, as set out in our protocol for this study (O'Leary et al., 2022). This typology is repeated in Table 1.

**Table 1:** Typology of relevant interventions.

Study title	Details of treatment/control group intervention(s)	Mean age (SD)	Sex	Sample size	% Homeless	Complexity of needs
Burnam (1996)	Treatment Arm 1 (*n* = 67): a social model residential programme providing integrated mental health and substance abuse treatment; Treatment arm 2 (*n* = 144): a community‐based non‐residential programme using the same social model approach; Control (*n* = 65): opportunity to access existing available community services (such as homeless shelters, a mental health clinic, a day socialisation centre, and AA groups).	37	Male: *n* = 231 (84%) Female: *n* = 45 (16%)	276	100	Mental Health Issues; Substance Abuse
Collins (2019)	Treatment (*n* = 86): HaRT‐A which is an application of motivational interviewing within a harm‐reduction treatment context; Control (*n* = 83): attended assessment sessions at each time point and had access to outreach; case management; nursing/medical care; access to external service providers; and assistance with basic needs (i.e., food, clothing, income, housing).	47.86 (9.56)	Male: *n* = 128 (76%) Female: *n* = 41 (24%)	169	100	Substance Abuse
Lester (2007)	Treatment (*n* = 57): behavioural day treatment, abstinence‐contingent housing, and abstinence‐contingent vocational training; Control (*n* = 61): received same intervention EXCEPT behavioural day treatment and aftercare.	Treatment: 40.54 (7.35) Control: 40.02 (7.01)	Male: *n* = 88 (75%) Female: *n* = 30 (25%)	118	100	Mental Health Issues; Substance Abuse
Milby (2000)	Treatment (*n* = 56): behavioural day treatment plus abstinence contingent housing and work therapy; Control (*n* = 54): behavioural day treatment alone. Behavioural day treatment utilised objectively defined, long and short‐term participant goals which addressed five common domains of dysfunction: substance abuse, homelessness, unemployment, lack of non‐drug related social and recreational activities, and emotional‐psychiatric problems.	38.1 (7.4)	Male: *n* = 84 (76%) Female: *n* = 26 (24%)	110	100	Mental Health Issues; Substance Abuse
Milby (1996)	Treatment (*n* = 89): enhanced day treatment programme plus abstinent contingent work therapy and housing; Control (*n* = 87): usual care consisting of day treatment programmes (12 steps based, individual and group counselling, medical evaluation and treatment).	Treatment: 36.0 (6.6) Control: 35.7 (6.2)	Male: *n* = (79%) Female: *n* = 27 (21%)	176	100	Substance Abuse
Milby (2003)	Same study as Milby (2000).					
Milby (2010)	Same study as Lester (2007).				
Nyamathi (2017)	Treatment (*n* = 65): dialectical behavioural therapy‐case management (DBT‐CM) – group session to support avoiding and eliminating cues to use, burning bridges to substance use, mindfulness, and diary card/review of homework; Control (*n* = 65): one‐on‐one Health promotion sessions with the nurse or community health worker.	Treatment: 39.1 (11.5) Control: 38.6 (11.3)	Male: *n* = 0 (0%) Female: *n* = 130 (100%)	130	100	Substance Abuse
Reback (2010)	Treatment (*n* = 64): Contingency Management (CM) was implemented in a community HIV prevention setting and targeted reduced substance use and increased health‐promoting behaviours; Control (*n* = 67): control group also earned points for attending scheduled study visits and participating in the HIV prevention programme activities.	36.4 (8.7)	Male: *n* = 131 (100%) Female: *n* = 0 (0%)	131	100	Substance Abuse; Physical Health Issues
Slesnick (2020)	Treatment (*n* = 75): Cognitive Therapy for Suicide Prevention (CTSP) provided in 10 (50 min) session protocol including weekly or bi‐weekly meetings focussing on education around cognitive processes, and implementation of cognitive restructuring and behavioural change; Control (*n* = 75): Drop‐in centre and activities open to control group, with access to therapy that (unlike the experimental suicide prevention intervention) are unsystematic and not manualized.	20.99 (1.96)	Male: *n* = 89 (59.3%) Female: *n* = 61 (40.7%)	150	100	Mental Health Issues
Thompson et al. (2017)	Treatment (*n* = 30): based on three stylistically consistent and easily integrated theoretical frameworks: Motivational Interviewing (MI), Transtheoretical Model (TTM), and the Information‐Motivation‐Behavioural Skills (IMB) Model; Control (*n* = 31): provided similar information on alcohol and sexual risk without the active theoretical components.	19.28 (1.20)	Male: *n* = 25 (41.67%) Female: *n* = 36 (58.33%)	61	100	Substance Abuse; Physical Health Issues
Thompson et al. (2020)	Treatment (*n* = 20): (a) brief daily technology‐supported self‐monitoring of alcohol, marijuana, and sexual risk behaviours (2–3 min/day) over 28 days and (b) brief motivational sessions at Weeks 0, 2, and 4 to promote use of OnTrack, encourage risk reduction, and provide graphed personalised feedback from the self‐monitoring data; Control (*n* = 20): included two components: (a) substance use treatment and referral and HIV testing, as regularly offered to all participants who report substance use and sexual risk behaviours at the shelter, and (b) brief meetings (20 min or less) with a research coordinator every 2 weeks.	19.1 (0.81)	Male: *n* = 28 (70%) Female: *n* = 12 (30%)	40	100	Substance Abuse; Physical Health Issues
Garland et al. (2017)	Treatment Arm 1 (*n* = 64): MORE unites complementary aspects of mindfulness training, third‐wave cognitive‐behavioural therapy, and principles from positive psychology into an integrative intervention strategy; Treatment Arm 2 (*n* = 64): CBT provided training in cognitive, behavioural, and interpersonal coping skills on the following topics: safety; PTSD: taking back your power; detaching from emotional pain; when substances control you; compassion; honesty; recovery thinking; setting boundaries in relationships; healing from anger; termination; Control (*n* = 52): participation in a therapeutic milieu; psychoeducation on topics related to addiction; client‐centred, supportive‐expressive group therapy; and coping skills groups.	Treatment Arm 1: 37.7 (10.4) Treatment Arm 2: 36.5 (11.2) Control: 38.7 (9.8)	Male: *n* = 116 (100%) Female: *n* = 0 (0%)	116	100	Mental Health Issues; Substance Abuse
Upshur (2015)	Treatment (*n* = 42): training and supports to the medical leadership and Primary Care Providers (PCPs), modifying the electronic medical record (EMR), and training for Care Managers (alcohol education materials, ongoing self‐management support, linkage to formal addiction treatment services and self‐help groups, and wellness counselling and goal setting); Control (*n* = 40): received usual care for medical conditions, including any behavioural health or drug or alcohol use problems, but did not have access to intervention PCP or CM support.	Treatment: 44.8 (8.4) Control: 46.0 (10.5)	Male: *n* = 0 (0%) Female: *n* = 82 (100%)	82	100	Mental Health Issues; Physical health Issues; Substance Abuse
Johnson (2011)	Treatment (*n* = 35): offered biweekly CBT sessions focussing on psychoeducation (regarding interpersonal violence, PTSD, safety planning, and empowerment) and CBT skills to manage PTSD and its associated features (e.g., cognitive restructuring, self‐soothing, assertiveness training); Control (*n* = 35): received standard shelter services (SSS) including case management, a supportive milieu environment, and attendance of educational groups offered through the shelter (no therapy was offered through SSS).	32.55 (8.00)	Male: *n* = 0 (0%) Female: *n* = 70 (100%)	70	100	Mental Health Issues
Tucker et al. (2017)	Treatment (*n* = 100): motivational interviewing approach that consisted of four distinct 45‐min sessions that rotated on a weekly basis throughout the 16‐week period. Topics focussed on alcohol and drug use (and their effects), sexual health and wellbeing, STI control and prevention, and development of coping strategies; Control (*n* = 100): received ‘usual care’, which included access to all of the basic services (e.g., food, hygiene), case management, and programmes that were available at the drop‐in centre at the time of the study.	21.8	Male: *n* = 146 (73%) Female: *n* = 54 (27%)	200	100	Mental Health Issues; Physical health Issues; Substance Abuse
Washington (2009)	Treatment (*n* = 40): 90 min of Life Management Enhancement (LME) cognitive‐behavioural group therapy twice a week for six weeks (12 sessiONS), also received one session of ‘touch therapy’ which included massage therapy; Control (*n* = 36): participated in one of six non‐LME groups that were held at the same time during the 6‐week period as the LME groups.	Treatment: 52.90 (3.46) Control: 54.75 (5.62)	Male: *n* = 0 (0%) Female: *n* = 76 (100%)	76	100	Mental Health Issues
Collins (2021)	Treatment Arm 1 (*n* = 74): HaRT‐A plus intramuscular injections of 380 mg extended‐release naltrexone – Hart‐A uses a compassionate, pragmatic, and participant‐driven approach to reducing participant alcohol intake without a focus on abstinence achieved through regular testing and tracking of intake and related discussions (lead by physicians or nurses) about better drinking strategies; Treatment Arm 2 (*n* = 78): HaRT‐A plus placebo injection; Treatment Arm 3(*n* = 79): HaRT‐A alone.; Control (*n* = 77): community‐based supportive services including emergency shelter or permanent supportive housing; intensive case management; basic nursing or medical care; referral to external service providers; and assistance with basic needs.	Treatment Arm 1: 49.27 (9.11) Treatment Arm 2: 46.55 (10.46) Treatment Arm 3: 49.38 (7.35) Control: 47.51 (9.50)	Male: *n* = 258 (86%) Female: *n* = 50 (14%)	308	100	Mental Health Issues; Physical health Issues; Substance Abuse
Kashner (2002)	Treatment (*n* = 111): contingency management approach where participants were rewarded with work opportunities (continued employment, higher wages, more hours, more responsibilities) based on client work behaviour (punctuality, productivity, reliability) and health behaviour (sobriety and use of advised assistance programmes); Control (*n* = 31): access to comprehensive rehabilitation, addiction, psychiatric and medical services.	Treatment: 42.7 (7.2) Control: 44.4 (7.6)	Male: *n* = 142 (100%) Female: *n* = 0 (0%)	142	100	Mental Health Issues; Physical health Issues; Substance Abuse
Koffarnus et al. (2011)	Treatment Arm 1 (*n* = 43): contingency management offering paid job skills training for those abstaining from alcohol; Treatment arm 2 (*n* = 42): offered paid job skills training with no abstinence contingencies; Treatment Arm 3 (*n* = 39): offered unpaid job skill training with no abstinence contingencies. (no control group),	Treatment Arm 1: 42.0 (8.5) Treatment Arm 2: 45.2 (8.0) Treatment Arm 3: 43.0 (7.6)	Male: *n* = 100 (81%) Female: *n* = 24 (19%)	124	100	Mental Health Issues; Substance Abuse
De Leon (2000)	Treatment Arm 1 (*n* = 138): therapeutic community approach which focussed on peer self‐help and the community as both the context and agent for change (i.e., using community‐as‐method) and was adapted to mentally ill chemical abuser (MICA) clients in three critical ways: increased flexibility, less intensity, and greater individualisation; Treatment Arm 2 (*n* = 93): also a therapeutic community approach but with fewer demands on clients and more flexible in accommodating individual needs and deficiencies; Control (*n* = 66): support offered included general residential programmes and other supported housing programmes, with or without day treatment services, those receiving case management services, as well as those discharged to self or to another family member, with or without follow‐up.	35.35 (6.89)	Male: *n* = 256 (75%) Female: *n* = 86 (25%)	342	100	Mental Health Issues; Substance Abuse
Himle (2014)	Treatment (*n* = 29): received eight, 2‐h sessions Work‐related Cognitive Behavioural Therapy Sessions (WCBT), held twice weekly over the course of four weeks in addition to standard vocational services. WCBT sessions included psychoeducation, cognitive restructuring, and exposure exercises, sometimes including social skills training related to the work environment; Control (*n* = 29): received standard vocational services which included, but were not limited to, career assessment, résumé construction, job interviewing skills, and job placement assistance.	43.59 (8.48)	Male: *n* = 39 (67.2%) Female: *n* = 19 (32.8%)	58	100	Mental Health Issues; Substance Abuse
Rew (2022)	Treatment Arm 1 (*n* = 150): received pre‐test & Intervention. Intervention consisted of six psychoeducational modules delivered one‐on‐one informed by a motivational interviewing practice and positive psychology. The modules focused on factual information and skill development such as assertive communication, general goal setting, condom use, and refusal of risky behaviour to promote their health and avoid or decrease their use of substances such as alcohol and other drugs and increase their safer sexual behaviours; Treatment Arm 2 (*n* = 151): Intervention only (see above); Treatment Arm 3 (*n* = 151): Pretest & Control: received health and social services from drop‐in centres; Control (*n* = 150): see above.	No Information	No Information	602	100	Mental Health Issues; Physical health Issues; Substance Abuse
Milby et al. (2008)	Same study as Lester (2007)					
Petry et al. (2000)	Treatment (*n* = 19): contingency management approach entered participants into a draw for reward slips for successfully completing group and individual goals. Goals ranged from group goals of reduced alcohol or drug intake, to individual goals such as joining a support group such as AA, and slips included small (e.g., $1 coupons), medium (e.g., $20 value gifts such as watches, backpacks, etc.) and large gifts (e.g., handheld TV); Control (*n* = 23): 4 weeks of an intensive outpatient day programme and then transfer to aftercare including 12‐step‐oriented spiritual reading, other group meetings focusing on life skills training, relapse prevention, coping skills training, AIDS education, social/recreational training, and 12‐step‐oriented meetings (as well as employment counselling).	Treatment: 47 (SE 2) Control: 48 (SE 2)	Male: *n* = 42 (100%) Female: *n* = 0 (0%)	42	Treatment: 53 Control: 48	Mental Health Issues; Physical health Issues; Substance Abuse
Tracy et al. (2007)	Treatment (*n* = 15): used Petry et al. (2000) low‐cost contingency management approach (see above); Control (*n* = 15): had access to all shelter services for the duration of the study.	No Information	No Information	30	100	Mental Health Issues; Substance Abuse

#### 2.2.2 Psychosocial interventions and adults experiencing homelessness

Psychosocial interventions are often used to address problematic substance use, poor mental health, and offending behaviours, as well wider social determinants of health such as housing instability and homelessness, worklessness, and poor skills or education. As adults experiencing homelessness will often deal with more than one of these issues at any given time, many will access services that use psychosocial interventions. It is therefore essential to understand whether these interventions are effective for adults experiencing homelessness.

#### 2.2.3 How the intervention might work

Broadly speaking, the main mechanism of change underpinning these interventions is psychological, focusing on the individual's psychological development and interaction with their social environment. However, there is no single theory of change underpinning these types of interventions; some are more explicitly based on formal theories, others less so. England et al. (2015) and others argue that psychosocial interventions draw on different theoretical models. In some areas, there are many different interventions derived from the same theoretical model. They also suggest that a number of interventions are adaptations of other interventions targeting different ages, delivery methods (e.g., individual, group), or settings. In developed the protocol for this study (O'Leary et al., 2022), the review team attempted to draw these varying models together into three overarching meta‐theories of change. In completing this review, we examined each of the included studies to identify whether these three meta‐theories were used, and more broadly, whether and how the study authors explained the likely causal mechanisms for the evaluated intervention. The results of this analysis are set out in Section 5 in relation to research question 2; we found no evidence that the meta‐theories were used, and many of the included studies simply referred to previous effectiveness research to justify the intervention design.

### 2.3 Why it is important to do the review

Psychosocial interventions increasingly play a role in policy responses to homelessness and the harms caused by homelessness. Yet while we do know about the effectiveness of some psychosocial interventions, there is no systematic review that is specific to adults experiencing homelessness. This proposed review will provide policy makers, commissioners, and service providers with insight into the effectiveness of different psychosocial interventions for this specific population.

There is also a significant gap in the extant effectiveness evidence in terms of the voices of people with lived experience of homelessness, which largely treats people with lived experience as passive research participants. This review aimed to elevate the voices of people with lived experience in two ways. First, there was an experts by experience review process that ran alongside the technical peer review process. This enabled the review team to gain views on the relevance and appropriateness of the definitions of homelessness and psychosocial interventions underpinning this review, the theories of change identified, and the outcomes used in the underlying studies. We held several workshops throughout the review to gain feedback from this group. Secondly, the team worked with a panel of people with lived experience to co‐produce the discussion, recommendations, and conclusions of the published review. This panel has been involved in the scoping work in preparing this protocol, through a workshop to discuss definitions of homelessness and psychosocial interventions, the review objectives, and how further to involve individuals with lived experience in the conduct of the review.

#### 2.3.1 Previous reviews

Luchenski et al. (2018) identified the absence of a systematic review on psychosocial interventions specific to the population of people experiencing homelessness. A recently published systematic review and meta‐analysis by Hyun et al. (2020) focused on the psychosocial outcomes of psychosocial interventions for adults experiencing homelessness. However, they focused on different outcomes to those covered by our review. A systematic review by Scott et al is currently under review, and focuses on psychosocial outcomes (which are not the focus of our review).

## 3 OBJECTIVES

The protocol for this review (O'Leary et al., 2022) established the following objectives for this review:

1. How effective are psychosocial interventions in the treatment of adults who are experiencing homelessness?
2. What are the explicit theories of change underpinning psychosocial interventions?
3. Are there differences in the effectiveness of psychosocial interventions in terms of their underlying theories of change?
4. Which type of intervention (e.g., talking therapies, behavioural incentives, self‐help) is most/least effective compared to treatment‐as‐usual?
5. Are there differences in the effectiveness of psychosocial interventions in terms of improving specific outcomes (e.g., housing stability, problematic substance use, mental ill health)?
6. For whom do the interventions work best?

## 4 METHODS

### 4.1 Criteria for considering studies for this review

We set out the methods used for this review in the published protocol (O'Leary et al., 2022).

#### 4.1.1 Types of studies

Eligible studies included in this review were impact evaluations with designs at levels, 3, 4 and 5 of the Maryland Scientific Methods scale [1], for example:

- *Level 3. Comparison of outcomes in treated group after an intervention, with outcomes in the treated group before the intervention, and a comparison group used to provide a counterfactual (e.g., difference in difference) … techniques such as regression and (propensity score matching may be used to adjust for difference between treated and untreated groups*.
- *Level 4. Quasi‐randomness in treatment is exploited, so that it can be credibly held that treatment and control groups differ only in their exposure to the random allocation of treatment. This often entails the use of an instrument or discontinuity in treatment, the suitability of which should be adequately demonstrated and defended*.
- *Level 5. Reserved for research designs that involve explicit randomisation into treatment and control groups, with RCTs providing the definitive example. Extensive evidence provided on comparability of treatment and control groups, showing no significant differences in terms of levels or trends*.

This therefore included all studies categorised as either ‘Randomised Controlled Trials’ or ‘non‐experimental designs with a comparison group’ from the studies which form the basis of the Homelessness Effectiveness Studies Evidence and Gap Map (EGM) 5th edition created by the Centre for Homelessness Impact (CHI) and the Campbell Collaboration (Jain et al., 2023).

Studies excluded included those with designs at levels 1 and 2 of the Maryland Scientific Methods scale, such as (1) studies without a control or comparison group; (2) ‘before versus after’ designs (without an untreated comparison group); and (3) cross‐sectional regressions.

As the review will therefore essentially included randomised and non‐randomised studies we undertook sensitivity analyses to investigate the effect of the inclusion of non‐randomised studies in the meta‐analysis.

[1] Level descriptions taken from https://whatworksgrowth.org/resources/the-scientific-maryland-scale/.

#### 4.1.2 Types of participants

There are a number of definitions of homelessness available, reflecting differences between countries and over time. There are also different forms of homelessness, taking into account the length of time someone has been experiencing homelessness, distinctions between living on the street or in their vehicles, or having a temporary place to stay.

We drew on the definition of homelessness used by Keenan et al. (2020) in a recently published Campbell Collaboration protocol. This definition was considered by a workshop of five individuals with lived experienced of homelessness, following which we slightly adapted and widened the definition. The definition we used for this review is:

‘Homelessness is defined as those individuals who are in inadequate accommodation (environments which are unhygienic and/or overcrowded), who are sleeping rough (sometimes defined as street homeless), those in temporary accommodation (such as shelters and hostels), those in insecure accommodation (such as those facing eviction or in abusive or unsafe environments), and people whose accommodation is inappropriate (such as those living in tents or vehicles, or “sofa surfing”)’.

Our focus was on adults (men and women aged 18 years and over), undertaken in any high‐income country and published in English. Studies of families or children were excluded from the review. In many countries (particularly the United Kingdom), there are different legal frameworks that apply to families and children experiencing homelessness, and thereby their access to different types of services, and different outcomes expected.

#### 4.1.3 Types of interventions

Given the varying (and often broadly scoped) definitions of what constitutes psychosocial interventions, and the significant differences in whether and how these interventions are structured and delivered, it is important that we be clear about the types of interventions we covered in this review.

The review focused on formal psychosocial interventions used with adults experiencing homelessness, or where at least 40% of the sample was adults experiencing homelessness. Interventions based solely or mainly on pharmacological approaches were excluded, as were interventions that might be expected to result in a psychosocial outcome, but are not formally recognised as being psychosocial interventions. The National Institute for Health and Care Excellence (NICE) provides some help here, stating that formal psychosocial interventions include: contingency management, behavioural couples therapy, community reinforcement approach, social behaviour network therapy, cognitive behavioural relapse prevention‐based therapy, and psychodynamic therapy (NICE, 2007). We used this to develop a typology of psychosocial interventions to help focus this review. The typology (Table 1) was discussed and validated with an expert panel of academics, policy makers, experts by experience, and practitioners involved in psychosocial interventions targeted at people experiencing homelessness, held in November 2021. This expert panel was convened by the CHI as part of the scoping work undertaken to develop this protocol. The primary purpose of the typology was to categorise studies for eligibility purposes, and to structure the analysis of the effectiveness of individual interventions.

The typology categorises specific interventions as either low intensity or high intensity, drawing on the distinction made by the Welsh Government (2011) between interventions normally delivered as a single session, and interventions that are formal and structured and delivered over a number of sessions. If feasible, we propose to see whether intensity is an important variable in the effectiveness of psychosocial interventions for adults experiencing homelessness.

The typology further categories interventions by their type. Talking therapies are a type of psychosocial intervention that primarily involves the service user discussing issues around their thoughts, feelings, or behaviours with a professional therapist. These interventions might be delivered in group or one‐to‐one settings.

Behavioural incentives are a type of psychosocial intervention that use extrinsic rewards or negative consequences to change an individual's behaviour. Finally, self‐help interventions are a group of psychosocial interventions in which individuals work through therapeutic materials or processes on their own, or with minimal input from a professional therapist. This can involve working in a group with others also going through the same process. Again, if feasible, we intend to examine whether intervention type is important in terms of the effectiveness of psychosocial interventions (Table 1).

#### 4.1.4 Types of outcome measures

The review will focus on three outcomes associated with psychosocial interventions and which are directly relevant to adults experiencing homelessness. These outcomes are: (1) Housing instability; (2) Problematic substance use; and (3) Mental ill health. We have not examined psychosocial outcomes, as these are covered by a published systematic review (Hyun et al., 2020) and because many people experiencing homelessness face a ‘tri‐morbidity’ of homelessness, substance use, and mental ill health (Cornes, Whiteford, et al., 2018). As these are often the three most significant issues facing people experiencing homelessness, it is appropriate to focus this review on whether these interventions generate change in these outcome areas. The review investigated these outcomes, primarily identifying studies by outcome from 594 studies which are the basis of the Homelessness Effectiveness Studies EGM 5th edition created by the CHI and the Campbell Collaboration (Jain et al., 2023). These outcomes are measured in a number of ways by primary studies. To have been included, a study must have measured changes in outcomes in at least one of these three outcome areas.

As such, we expected a range of continuous and binary outcomes to feature in the reviewed studies, and we converted these into the same metric (e.g., Hedges' *g*) for meta‐analysis (Borenstein et al., 2009). Where effect sizes are converted from a binary to continuous measure (or vice versa, depending on our ultimate choice of effect size), we undertook sensitivity analysis to investigate the effect of the inclusion of studies with a converted effect size in the meta‐analysis.

#### 4.1.5 Duration of follow‐up

We incorporated post‐test measurements and up to two additional follow‐up time points in their analysis. In cases where there were more than two follow‐up points available, the reviewers selected the time points closest to 3 months and 12 months. To evaluate the impact of different follow‐up durations, a sensitivity analysis was conducted comparing the relative effects of short‐term (0–6 months) and long‐term (6–12 months) follow‐up periods. This analysis was contingent upon the availability of sufficient studies with reported data for both time frames.

#### 4.1.6 Types of settings

Our inclusion criteria encompassed studies conducted in any type of setting that utilised psychosocial interventions. However, we specifically included studies conducted exclusively in high‐income countries. This decision acknowledges the substantial disparities between high‐ and low‐income countries regarding access to resources, services, and the underlying factors contributing to homelessness (Magwood et al., 2020).

### 4.2 Search methods for identification of studies

#### 4.2.1 Electronic searches

The primary method employed to identify studies for this review was through the utilisation of the Effectiveness EGM 5th edition, developed and published by The Campbell Collaboration (Jain et al., 2023). This edition of the EGM incorporates searches completed in August 2022.

The EGM focuses on effectiveness studies, in the form of systematic reviews and impact evaluations. It shows relevant evidence organised into an interactive online matrix capturing where there is evidence for different categories of intervention and how they affect a range of outcomes. The Effectiveness EGM provides the initial search from which studies for this review were selected. Specifically, eligible studies were those with designs at levels, 3, 4 and 5 of the Maryland Scientific Methods scale, as previously described.

The process for identifying and searching for the studies included in the EGM list is described by White et al. (2019) in their published *PROTOCOL: Studies of the effectiveness of interventions to improve the welfare of those affected by, and at risk of, homelessness in high income countries: An evidence and gap map*. The EGM's authors undertook a systematic search of a number of academic databases, evidence and gap map databases, systematic review databases, and trial registries. They also undertook grey literature and website searches. The search terms used were published in both the protocol and are replicated in Supporting Information S1: Appendices to this article. The authors state that all titles and abstracts, and then full text, were double screened, with a third‐party arbitrator in the event of disagreement. The authors also contacted the authors of each included study, providing a copy of the draft version of the EGM for comment. The authors make clear their data extraction and coding strategy, stating that coding was done independently by two coders, with a third‐party arbitrator in the event of disagreement. Finally, the authors state that included primary studies were subjected to critical appraisal using Campbell's Critical Appraisal Tool for Primary Studies, and for included systematic reviews using AMSTAR 2. At least two other systematic reviews that utilise the EGM as the primary sources for searches have been published by Campbell Systematic Reviews (O'Leary et al., 2024; Weightman et al., 2023).

#### 4.2.2 Searching other resources

In January/February 2022, the review team issued a call for grey evidence (with a deadline of 28th February 2022) which was disseminated through Manchester Metropolitan University and the CHI social media channels, inviting people with lived experience, researchers, commissioners, service providers and wider stakeholders to submit relevant grey evidence for consideration in the review. Specifically, the call was for evidence that is:

- empirical, based on research that:
  o elevates the voice to people with experience of homelessness;
  o measures the impact of interventions (before and after, quasi‐experimental, RCT);
  o identifies the barriers to, and facilitators of, successful implementation of interventions;
  o is about psychosocial interventions with outcomes around housing instability, problematic substance use, and mental ill health;
  o is not published in a book or academic journal; and
  o is specific to the United Kingdom, or England, Northern Ireland, Scotland or Wales.

The reviewers also hand searched key journals, using similar search terms and date ranges as White et al. (2019). While some may have already been searched as part of the EGM (Jain et al., 2023), this targeted journal search and more substance use and treatment focused search was undertaken to further ensure the capture of all existing literature and evidence. These journals were chosen in consultation with the CHI and other experts in the evidence base around homelessness. The hand searched journals were:

- *Psychiatric Services Journal.*
- *American Journal of Public Health.*
- *European Journal of Homelessness.*
- *Housing Studies.*
- *Social Policy and Administration.*
- *Journal of Social Distress and Homelessness.*

#### 4.2.3 Description of methods used in primary research

Primary research included were based on designs at levels 3, 4 and 5 of the Maryland Scientific Methods scale, including experimental (randomised) and quasi‐experimental studies. Such studies measured the effectiveness of an intervention designed to reduce problematic substance use, housing instability, and/or mental ill health against another intervention or a control group (e.g., no intervention, treatment as usual, wait‐list).

#### 4.2.4 Screening

Studies were selected from the 5th edition of the EGM (Jain et al., 2023). Further studies were added to the shortlist from three sources:

- unpacking relevant systematic reviews contained in the EGM list;
- the call for grey evidence; and
- hand searches of key journals.

The reviewers started by undertaking title and abstract screening of each individual study: (a) listed in the Effectiveness EGM; (b) identified through the call for evidence; and (c) identified through hand searches. This title and abstract screening was undertaken independently by three reviewers, and any disagreements were escalated for adjudication to a subject matter expert or methods expert on the review team.

Systematic reviews from the EGM that are identified as being relevant (i.e., met the inclusion criteria used for this review) at title and abstract stage were unpacked. This list of unpacked studies was checked against the Effectiveness EGM, and any duplicates were removed. The remaining studies (i.e., studies included in relevant systematic reviews that do not appear in the Effectiveness EGM) were subjected to a title and abstract search against the review's inclusion and exclusion criteria. Again, the title and abstract screening of was conducted by three reviewers independently, and any disagreements were escalated for adjudication to a subject matter expert or methods expert on the review team.

Once the title and abstract screening was complete, each potentially includable study was full text screened using the inclusion/exclusion criteria previously defined. All full text screening was undertaken by two reviewers, and any disagreements were escalated to a subject matter or a methods expert on the review team. Twenty‐five percent (25%) of final screening decisions were sampled by a third reviewer. Final decisions about inclusion were made by all members of the review team, and a list of studies eligible for inclusion in this systematic review was agreed.

#### 4.2.5 Data extraction and management

Data extraction from eligible studies was conducted by two reviewers, who collected information on the study, quantitative data necessary for meta‐analysis, and assessments of each study's findings with respect to their confidence level (which were undertaken by the EGM authors using the Campbell Collaboration's critical appraisal tool for primary studies [White et al., 2019]). Specifically, the reviewers extracted:

- Publication details (e.g., authors, year, source, study location).
- Intervention details.
- Theory of change classification.
- Intervention classification.
- Participant details, including classification (e.g., age, gender).
- Study design.
- Comparison (e.g., other intervention, treatment as usual, waitlist control).
- Outcome description, definition and measurement (including measurement duration).
- Sample sizes of treatment and control groups.
- Data to calculate odds ratios or standardised mean difference (SMD).
- Confidence assessment.

Any discrepancies in coding were discussed and referred to the lead reviewer for resolution.

For studies obtained from the EGM we used the critical appraisal results provided by the EGM authors. The tool used to undertake these assessments is published (White & Narayanan, 2021). For eligible studies identified through the additional searches outlined above, our review team used undertook assessments using the same tool as that used by Campbell. Two reviewers independently assessed each of the relevant included studies, and any discrepancies between these assessments were adjudicated by a third team member. The results of the critical appraisals were used in sensitivity analysis to show whether inclusion of low quality studies had an effect on the overall results. The appraisals were not used to exclude any studies from this review.

#### 4.2.6 Measures of treatment effect

The studies that were included in our analysis reported both continuous and dichotomous outcome measures. We computed standardised effect sizes using the David‐Wilson Practical Meta‐Analysis Effect Size Calculator (Wilson, 2017). To calculate the standardised effect sizes, we extracted statistical information from the included studies, which varied based on the type of outcome measure. For continuous data, we used means and standard deviations, means and standard errors, unstandardised regression coefficients and pooled standard deviations, as well as results from *f*‐tests and *t*‐tests. For binary outcomes, we used binary proportions and 2 by 2 frequency tables to calculate odds ratios and risk ratios.

One of the papers included in our analysis (Burnam et al., 1995) only reported means without standard deviations or any other statistic that could be used to calculate standardised effect sizes. Similarly, another study (Milby et al., 1996) only provided *p*‐values. In the case of De Leon et al. (2000), only beta coefficients and *p*‐values were reported from an OLS regression, making it impossible to compute effect sizes. As a result, we have presented these studies narratively and excluded them from any meta‐analysis.

Another study (Upshur et al., 2015) reported medians without interquartile ranges when presenting outcomes in relation to monthly alcohol consumption, and these could not be included in our assessment of substance use outcomes. Therefore, when computing substance use effect sizes, we relied only on results presented on monthly illegal drug consumption.

Most of the papers included in our analysis reported outcomes based on a continuous scale, which – in line with the protocol – led us to use the SMD as the main effect size metric for our meta‐analyses with its 95% confidence interval. To correct for any small sample bias, we employed Hedges' *g* within the SMD. However, some studies reported binary outcomes for which we calculated log odds ratios. We decided on a case‐by‐case basis whether to convert the odds ratio to an SMD to combine them in a single meta‐analysis, taking into account whether the binary outcome had been derived from a continuous scale, such as blood alcohol level. Whenever we did make such a conversion, we relied on the assumptions outlined in the Cochrane handbook (Higgins et al., 2022). In cases where we could not convert the odds ratio to an SMD, we presented the results narratively in Section 5.

We note that standardising continuous outcomes into SMD, as described in the Cochrane handbook, does not adjust for differences in the direction of the scale between studies. Most of the outcome scales used in the included studies in our review indicated a lower score or number for improved outcomes (e.g., Addiction Severity Index; Brief Symptom Inventory), while others indicated better outcomes with a higher score or number (e.g., number of days housed in the last 60 days). To ensure that all scales pointed in the same direction, we multiplied the SMD and its corresponding confidence interval by −1 in the latter cases.

#### 4.2.7 Unit of analysis issues

We assessed for unit‐of‐analysis errors in the included studies in the review and identified the following potential sources of unit‐of‐analysis error:

- Multiple observations for the same outcome construct (i.e., measures at multiple follow‐up points are extracted; multiple measures of the same construct are extracted): all studies.
- Cross‐over trials: 1 study.
- Multiple trial arms of interest: 1 study.
- Cluster‐randomised trials (i.e., groups of individuals were randomised together to the same intervention): 3 studies.

When a study contributes more than one effect size to the meta‐analysis, (e.g., a study included more than two groups or measured an outcome using two or more instruments), the core assumption that each effect size in a meta‐analysis is independent is violated (Borenstein et al., 2009). To account for multiple follow‐up points and multiple measures being taken from the same study in the same meta‐analysis, we used Correlated and Hierarchical Effects (CHE) model (Pustejovsky & Tipton, 2022), which explicitly takes into account that some effect sizes within clusters are based on the same sample.

The included crossover trial (Tucker et al., 2017) did not publish the data required to include a paired analysis in a meta‐analysis that is suggested by Higgins et al. (2022) to avoid unit‐of‐analysis problem. Means and standard deviations were available only for measurements on intervention and control separately. Therefore, we have incorporated the trial in a meta‐analysis by taking all measurements from the two periods and analyse these as if the trial was a parallel‐group. Our decision is informed by the fact that the unit‐of‐analysis error might be regarded as less serious than some other types of unit‐of‐analysis error, given that this incorrect analysis is conservative (i.e., it results in studies being under‐weighted rather than over‐weighted; Higgins et al., 2022).

In one specific study, we encountered multiple sources of possible unit‐of‐analysis error: Garland et al. (2017) was a cluster‐randomised trial with multiple trial arms. First, to avoid a unit‐of‐analysis error due to clustering, we reduced the size of the trial to its effective sample size, by dividing the original sample size by a quantity called the ‘design effect’ (Rao & Scott, 1992). The design effect is approximately:

1+(−1)×ICC,

where M is the average cluster size and ICC is the intra‐cluster correlation coefficient. As ICC estimates were not reported, we used the estimates used in the study for power analysis that was derived from a meta‐analysis of cluster RCTs. Second, we combined the two intervention groups to avoid double‐counting and correlated effect sizes (Higgins et al., 2022) by pooling the mean and standard deviation of two trial arms before calculating a SMD using the pool.groups function in R.

One cluster randomised trial ignored group‐level random effects stating that ‘[a]ccounting for clustering by PCP did not substantially change estimates in the models, so results reported show only data by individual subject’ (Upshur, 2015, p. 22). Therefore we decided not to account for clustering.

For the one remaining cluster randomised trial (Himle et al., 2014), we did not make any corrections as their analytical approach accounted for clustering of observations.

#### 4.2.8 Dealing with missing data

Three papers (Burnam et al., 1995; De Leon et al., 2000; Milby et al., 1996) did not provide sufficient statistical information to calculate standardised effect sizes for some outcomes of interest in this review. Despite attempts to contact the authors for additional information, the necessary data was not obtained, resulting in the exclusion of these studies from statistical analyses. Findings of these studies are presented narratively in the appropriate Section 5. For Upshur et al. (2015), effect sizes were calculated for some relevant outcomes despite missing data.

#### 4.2.9 Assessment of heterogeneity

We presented standardised effect sizes and confidence intervals for each study (if possible) and visually examined forest plots for heterogeneity for each meta‐analysis. Since our data sets included multiple effect sizes clustered into studies, it was expected that effects from the same study would be more similar to each other than effects from different studies. Hence, we calculated *I* ^2^ using two variance components from the meta‐analytic outputs to determine the between‐cluster heterogeneity and within‐cluster heterogeneity. The *I* ^2^ values indicate the proportion of total variance attributable to the total amount of heterogeneity, including the sum of between‐ and within‐cluster heterogeneity.

As a random effects model is used in the analysis, we also present the prediction interval, which provides a more practical understanding of the possible range of effect sizes for each intervention by indicating the interval where approximately 95% of the true outcomes are estimated/predicted to fall (Borenstein et al., 2017).

#### 4.2.10 Assessment of reporting biases

To mitigate the possibility of publication bias, our search strategy included searches for grey literature (given that published studies are likely to report larger than average effects [Borenstein et al., 2009]). We also tested for the presence of publication bias through additional analysis, including funnel plots and Eggers tests (Egger et al., 1997) to determine whether the summary effects in the meta‐analysis are subject to publication bias, and if this appeared to be the case, further tests, such as Trim and Fill (Duval & Tweedie, 2000) were used to estimate the studies that might be missing and then added to the analysis to determine a ‘best estimate of the unbiased effect size’ (Borenstein et al., 2009, p. 286).

### 4.3 Data synthesis

Our final collection of studies and effect sizes included a variety of dependencies, which resulted in a complicated data set containing correlated effect sizes and sampling errors. Studies often reported effects for multiple measures of mental illness, housing instability, and substance use across multiple follow‐up times, or both. This variety of outcomes within studies suggested that there would be within‐study heterogeneity in effect sizes. In addition, we expected between‐study heterogeneity due to differences in intervention types with varying intensity and application timeframes. Some studies focused on specific subpopulations, such as suicidal youth, minority women, and alcohol‐dependent veterans. Furthermore, a few studies included multiple treatment arms compared to a common control group.

To handle the complexity of our data set, we employed the CHE model (Pustejovsky & Tipton, 2022) along with Robust Variance Estimation, using the metafor (Viechtbauer, 2010) and clubSandwich (Pustejovsky, 2022) packages in R. The CHE model, utilised in conjunction with Robust Variance Estimation, assumes that there is a consistent correlation among effect size groups from the same study, and permits both within‐study and between‐study heterogeneity in true effect sizes. Since we did not possess information on the probable correlation among effect sizes in our papers, we made an assumption of rho = 0.6 in all meta‐analyses.

Our meta‐analysis results are displayed on forest plots. For cases where we analyse a large number of dependent effect sizes within the same analysis, we present pooled estimates aggregated to the study level in the forest plots. This is because presenting the full set of individual effect sizes was not easily interpretable.

#### 4.3.1 Subgroup analysis and investigation of heterogeneity

We assessed whether there are sufficient studies to justify subgroup analyses and meta‐regressions, given that it is very unlikely that an investigation of heterogeneity will produce useful findings unless there is a substantial number of studies.

Our preferred approach was to include all our moderators of interest in the same analysis. However, given the inadequate number of studies and effect sizes, we estimated single variable meta‐regression models for some of our moderator of interest and interpret each of these with caution.

Using no‐intercept specification allowed us to assess and compare coefficients that represent average effect sizes for the corresponding category.

#### 4.3.2 Sensitivity analysis

We performed sensitivity analysis, when feasible, to examine if the outcomes of the meta‐analysis are affected by the exclusion of studies that have converted effect sizes from binary to continuous. Additionally, we evaluated the sensitivity of studies categorised as having low confidence by eliminating them from the meta‐analysis and comparing the outcomes to those obtained from the primary meta‐analysis.

## 5 RESULTS

### 5.1 Description of studies

#### 5.1.1 Results of the search

A total of 898 papers were screened at title and abstract stage. From the EGM searches, a total of 684 papers were reviewed. Additionally, 204 papers were identified from supplementary searches of relevant journals and systematic reviews, and 10 papers from hand search. After this screening stage, 814 papers were excluded, and 84 papers were progressed for full‐text screening.

**Figure 1:**
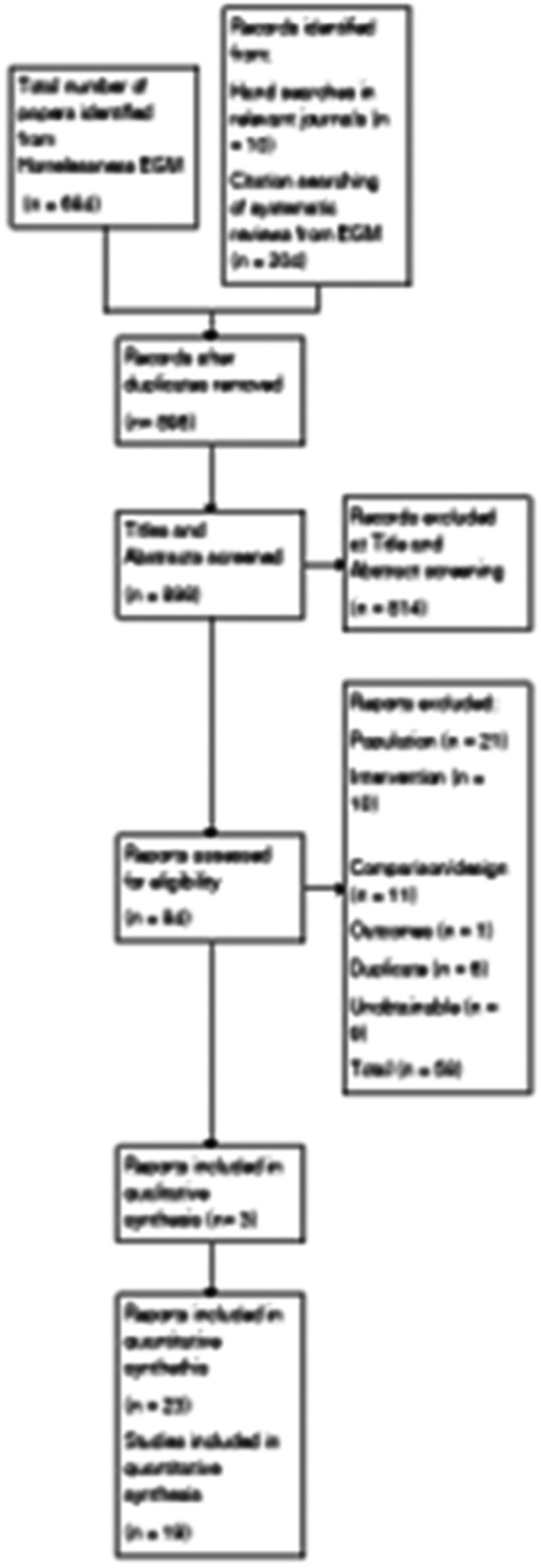
PRISMA flow diagram.

Out of the 84 papers sought for full review, 8 were unobtainable. Of the remainder, the most common reasons for exclusion were that the papers did not evaluate an intervention of interest as set out in our typology of interventions published in the review protocol (*n* = 9), did not target the population group of interest (*n* = 21), did not use an RCT or quasi‐experimental design (*n* = 29), or did not measure problematic substance use, housing, or mental health outcome (*n* = 1). After title and abstract screening, paper retrieval, and full text screening, 26 papers were included in the review, corresponding to 23 unique studies. More details can be found in the PRISMA diagram below (Figure 1).

#### 5.1.2 Included studies

Twenty‐six papers covering 23 studies were included in this review. All were conducted in the United States of America, and 22 of the studies were RCTs. One paper (one study), De Leon et al. (2000), was non‐randomised assignment to treatment and control groups. None of the included studies were systematic reviews. (We described above that relevant systematic reviews from the EGM were unpacked and their included studies assessed for inclusion in this review. In each case, the relevant systematic review included primary studies that were not relevant, or were duplicates of primary studies included in the EGM. Where individual studies met the inclusion criteria for this review, they were subject to full text review and critical appraisal undertaken.)

The included papers cover a total of *n* = 2296 participants; *n* = 1233 of whom were in treatment groups. Just under 36% of participants were women. Four of the papers focused only on women experiencing homelessness (Johnson et al., 2011; Nyamathi et al., 2017; Upshur et al., 2015; Washington & Moxley, 2009); and four focused only on men (Garland et al., 2017; Kashner, 2002; Petry et al., 2000; Reback et al., 2010). Four of the papers reported that participants were predominantly (90% and over) African Americans (Himle et al., 2014; Kashner, 2002; Milby et al., 2008, 2010; Washington & Moxley, 2009), and as mixed ethnicities in 18 papers. The population age also varies across the studies, with five studies focusing on young adults (aged between 17 and 25 years old), while the remaining studies have an age range of 18–80 years old, with a mean age of participants ranging from 33 years to 53 years old.

#### 5.1.3 Excluded studies

Details of the papers excluded at full text review, and the primary reason for exclusion, are provided in Supporting Information S2: Appendix.

### 5.2 Risk of bias in included studies

**Figure 2:**
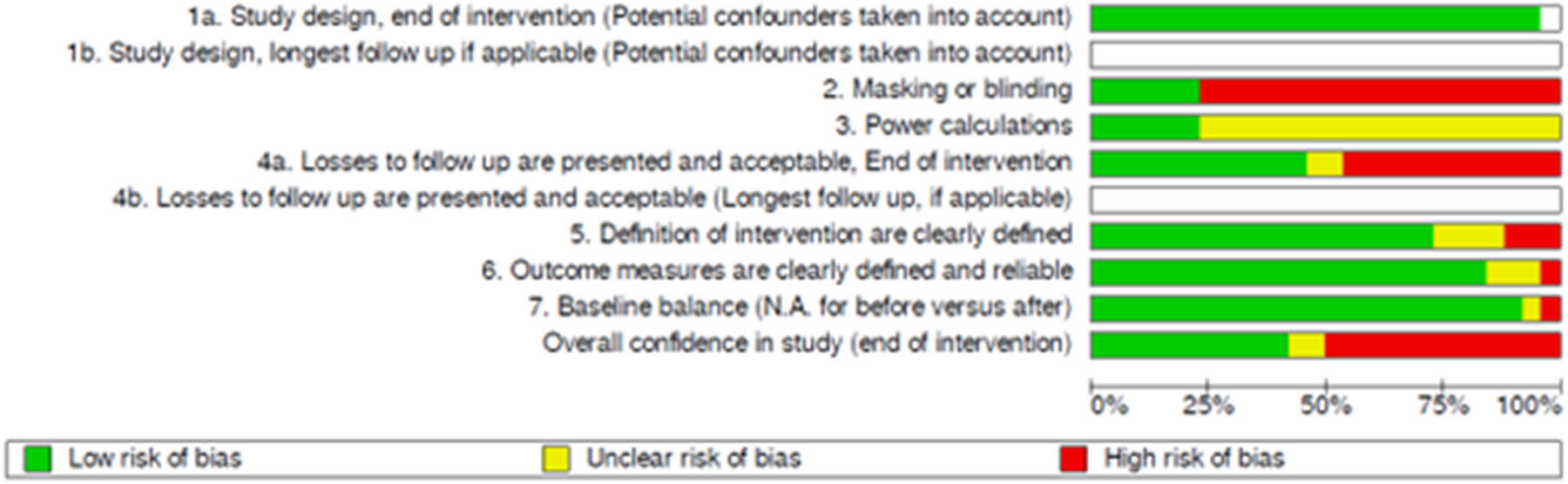
Summary of risk of bias assessments.

For 18 or the 26 included papers, risk of bias assessment was undertaken by the Campbell Collaboration using its critical appraisal for primary studies tool (White et al., 2019). For the remaining papers, we undertook the Risk of Bias assessments using this same tool, as outlined in Section 4 of this report. The overall results of these assessments in provided in Figure 2.

As all but one of the papers included in the review were RCTs, they were all rated as having high confidence on the use of a study design that accounted for confounding factors (*n* = 25). Most of the papers (*n* = 20) did not provide information on use of power or sample size calculations to calculate sample sizes. The majority of the papers (*n* = 20) did not report using masking/blinding of outcome assessors or the intervention allocation during analysis, resulting in a low rating for this criterion. Many of the papers included reported high rates of attrition (overall and differential between intervention and control) of participants. At the follow‐up point, 11 out of 27 papers were rated as low confidence due to the loss to follow‐up, 2 were rated as medium confidence, and 13 were rated as high confidence.

Regarding the reliability and validity of measures, as well as baseline balance of participants, the vast majority were rated as high confidence, with 20 of the paper providing clear descriptions of the intervention being tested (*n* = 19) and 23 of the papers using either validated outcome measures for problematic substance use, mental health, and housing, or providing sufficient description of related measures (*n* = 22). However, three papers were only rated as medium confidence on the outcome measures criterion due to a lack of clear information on the measure's validation and reliability, and one paper was regarded as low confidence. This paper (Lester et al., 2007) was a secondary analysis of another data set (Milby et al., 2008), and therefore the lack of reporting within the paper was not seen as an issue due to sufficient reporting in the parent study. Additionally, 25 out of the 26 papers reported balance on observable characteristics at baseline, indicating high confidence in this criterion.

**Figure 3:**
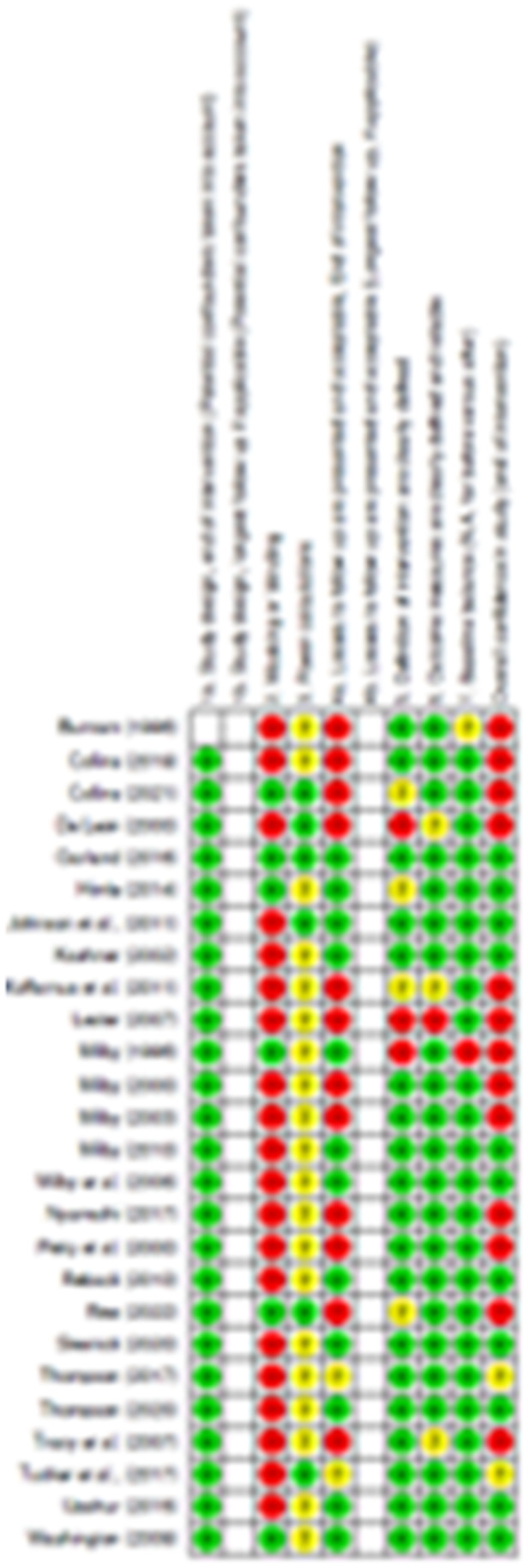
Study‐level risk of bias assessments.

The risk of bias assessment summary for each included study is provided below (Figure 3).

### 5.3 Synthesis of results

#### 5.3.1 RQ1: How effective are psychosocial interventions in the treatment of adults who are experiencing homelessness?

To answer our first research question, we looked at studies that compared any intervention in our typology to a control group. We investigated the average impact of all studies regardless of outcome type and we also looked at treatment effects for each outcome cluster separately.

##### All studies

**Figure 4:**
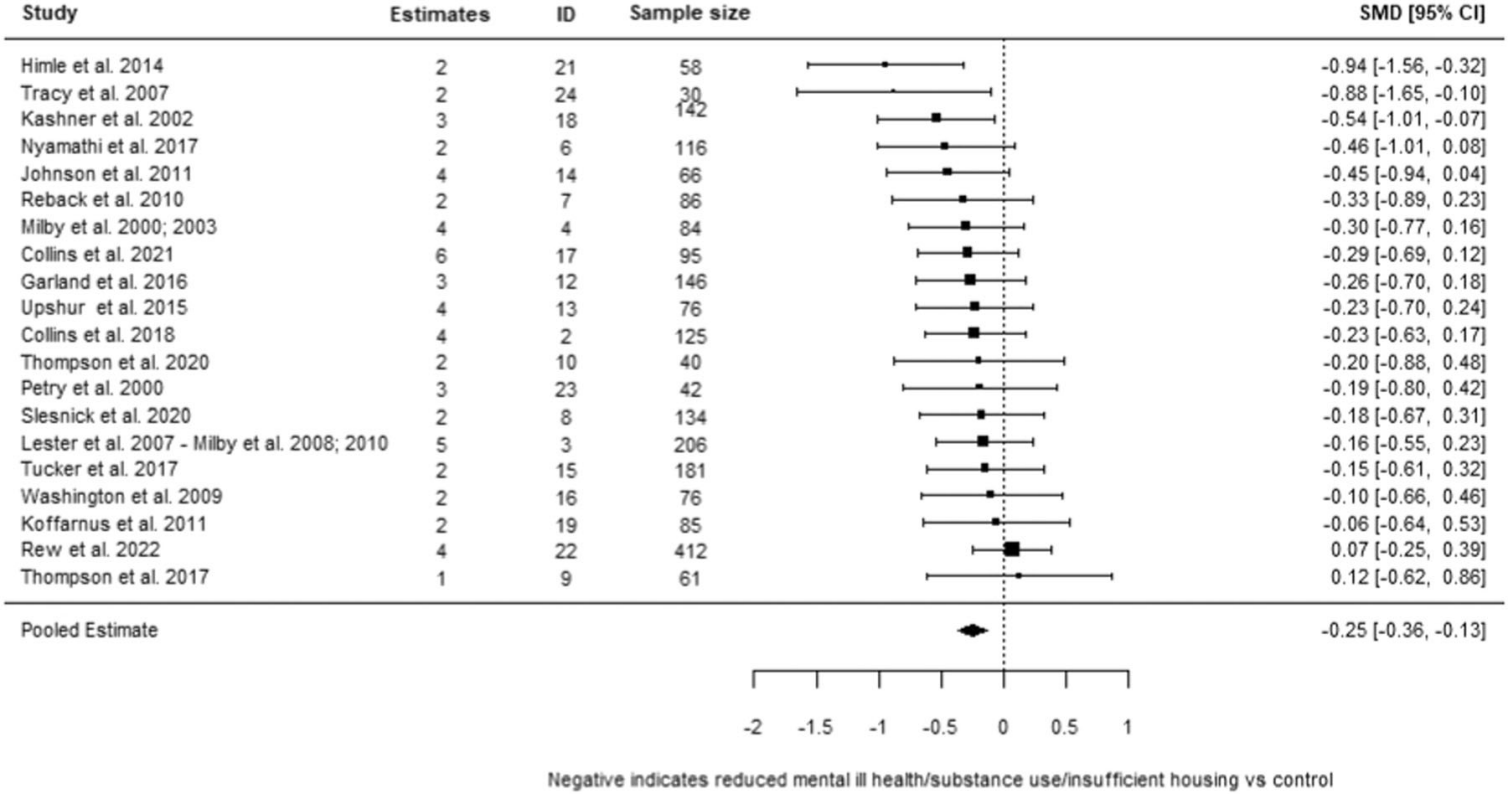
Effectiveness of psychosocial interventions.

Out of 26 included papers (corresponding to 23 studies), we were able to combine 59 effect sizes from 20 studies reported in 23 papers, using a CHE meta‐analysis with cluster robust estimation. Figure 4 below shows the meta‐analysis results to answer research question 1 when all individual analysis points are included irrespective of the type of outcome cluster.

Please note that we have averaged the individual effect size from each study for the purposes of displaying on the forest plot for this specific analysis as there were too many individual effects to display clearly. Estimates refer to the number of effect sizes included for each study in the meta‐analysis. The mean number of effect sizes per study was 2.95 with the number of effects ranging from 1 to 6. Multiple effect sizes from single studies resulted from multiple follow‐up periods, multiple outcome constructs and multiple measures of the same construct (e.g., mental ill health). All of the included studies were from the United States. They tested a range of types of interventions from our typology, covering Motivational Interviewing (MI), CBT, Contingency management (CM), Dialectical Behaviour Therapy (DBT), Brief Intervention (BI), and Brief Motivational Intervention (BMI) against housing instability, problematic substance use and mental ill health outcomes. The sample size column indicates the total number of people involved in the study across intervention and control group at follow‐up. We calculated an average effect of (−0.25 SD, 95% CI [−0.36, −0.13]), indicating on average an improvement of problematic substance use, housing instability and mental ill health for people experiencing homelessness who participated in the included programmes compared to those in TAU (or other non‐psychosocial) service provision. However, there is substantial heterogeneity across studies, which can be observed by looking at the forest plot. We calculated prediction intervals, that is, the expected range of true effects in similar studies in future settings (IntHout et al., 2016). Prediction intervals contain a much larger range of treatment effects than the confidence intervals, with a range of values on both sides of the line of no effect (indicating possible reduced or increased problematic substance use/housing instability and mental ill health as a result of being involved in these types of programmes). We estimated these to be ranging between −0.85 and 0.35. This heterogeneity is to be expected given the range of interventions being tested, the range of outcomes used, the complex diversity of needs of the people involved across different studies and the mix of follow‐up periods.

The *I* ^2^ value in this context indicates how much of the total variance can be attributed to the total amount of heterogeneity, which is the sum of between‐ and within‐cluster heterogeneity. We calculated an *I* ^2^ value of 67% (*I* ^2^ = 67.40%), which means that around two‐third of the total variance can be attributed to between‐ or within‐study heterogeneity (with the remaining 33% coming from sampling variation).

We tested the sensitivity of the results of this meta‐analysis to (1) removal of low confidence studies and (2) removal of studies where an effect size had been converted from a binary to a continuous outcome. We found that the point estimate and confidence intervals were not sensitive to the removal of studies where an effect size had been converted from a binary to a continuous outcome (−0.25 SD, 95% CI [−0.37, −0.12] – 17 studies, 51 effect sizes). However, the results were sensitive to the removal of low confidence studies (−0.29 SD, 95% CI [−0.42, −0.16] – 12 studies, 32 effect sizes). This suggests that the findings are sensitive to the inclusion of lower quality studies, although unusually the average effect increases when we remove the lowest confidence studies.

We did not include Burnam et al. (1995), Milby et al. (1996) or De Leon et al. (2000) in this overall meta‐analysis:

- Burnam et al. (1995) compared a residential programme and a non‐residential programme that both used a similar approach to reduce problematic substance use and mental ill health, with a control group described as ‘receiving no intervention but free to access other community services’ (p. 112). A total of 276 individuals were randomly assigned between these two treatments (*n* = 67 to the residential programme, *n* = 144 to the non‐residential) and control. The authors found no significant difference between the two treatment groups, except those in the non‐residential programme fared better at 3 months in terms of housing stability. The authors also found no significant differences in outcomes between the treatment and control group.
- De Leon et al. (2000) compared two versiONS of a ‘modified’ therapeutic community with treatment as usual. A total of *n* = 342 individuals entered one of these three groups (*n* = 183 assigned to the first treatment group, *n* = 93 to the second, and *n* = 66 to the control group). The study examined 12 measures across five outcome domains. The authors conclude that the modified therapeutic communities were more effective than treatment as usual.
- Milby et al. (1996) evaluated an intervention comprises of day treatment, work, and housing, (*n* = 69 participants) which was compared to treatment as usual (*n* = 62 participants). Outcomes of interest included housing stability, alcohol use, drug use, and days employed. The authors found significant differences between the treatment and control groups for all but one of the outcomes (days employed).

Overall, the findings here suggest that the psychosocial interventions being evaluated were – on average – more effective than treatment as usual in improving relevant outcomes. A random new intervention – however – may have a positive, negative or null effect. This is not surprising, given the complex diversity of the included studies. To further investigate RQ1, we categorised the 59 effect sizes used for the previous analysis according to the outcome of interest and run three separate meta‐analyses to assess the impact of psychosocial interventions on outcomes related to either (1) mental ill health, (2) problematic substance use or (3) housing instability. We included all types of interventions identified from our typology. The studies are still diverse within each outcome cluster, in terms of intervention, follow‐up points and populations. Therefore, we interpret the meta‐analysis results that answer research question 1 on the effect of psychosocial interventions with some caution.

#### 5.3.2 Reduction in problematic substance use

**Figure 5:**
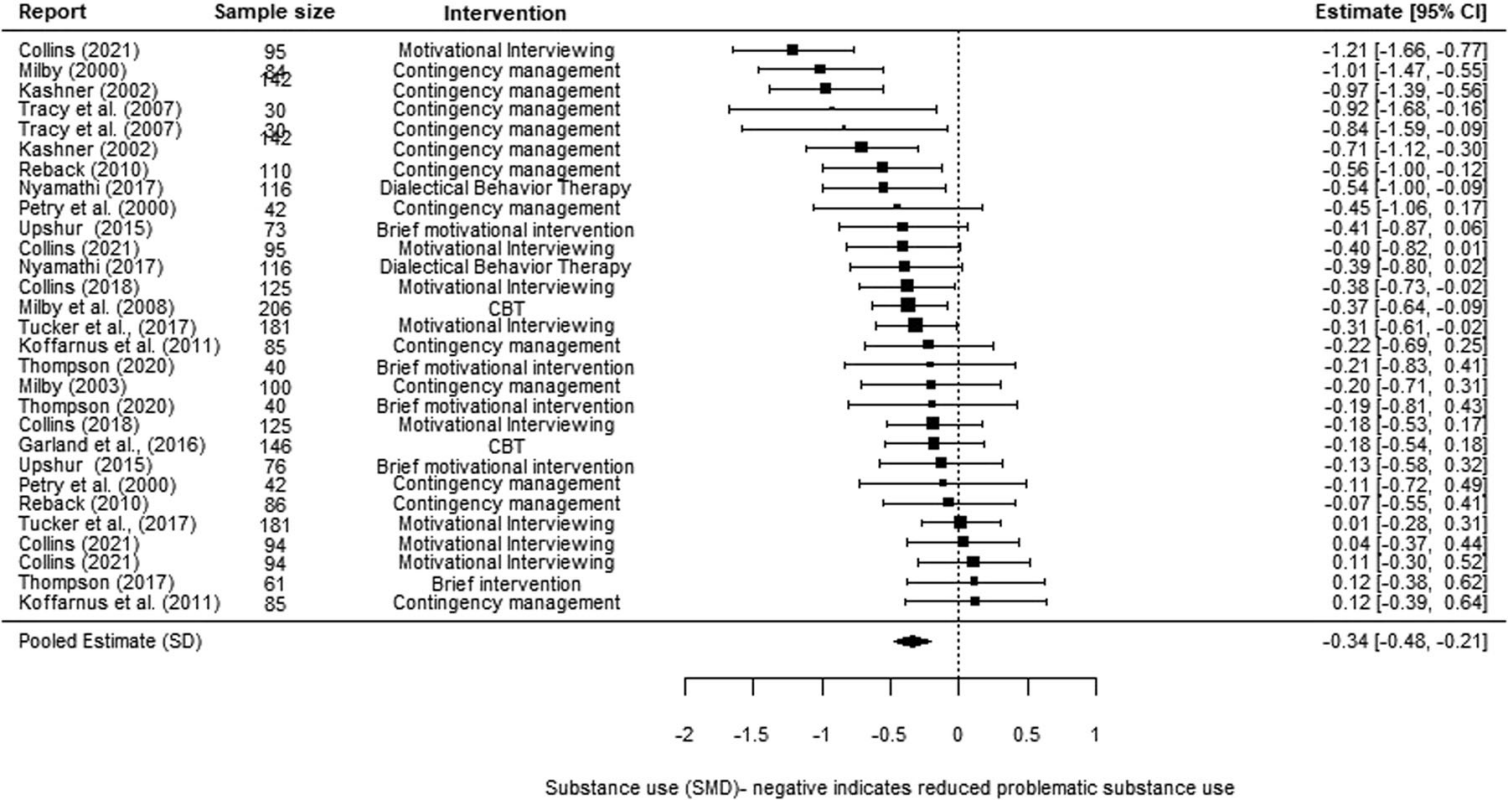
Effectiveness of psychosocial interventions on reducing problematic substance use.

We combined 29 effect sizes from 15 studies reported in 16 papers. The average effect for psychosocial interventions included in this study against problematic substance use is (−0.34 SD, 95% CI [−0.48, −0.21]). The prediction interval ranging from −1.00 to 0.31, with the fraction of variance that is due to heterogeneity is estimated to be 65% (*I* ^2^ = 64.66%). Figure 5 shows the results of the meta‐analysis for this outcome.

The sensitivity analysis suggests a robust estimation, with practically no substantive variation when excluding converted effect sizes from the analysis. The removal of low confidence studies, however, leads to a decreased average effect size estimate (−0.30 SD, 95% CI [−0.53, −0.07]).

Single variable meta‐regression models were also conducted separately for three moderator variables: intervention classification (behavioural incentives or talking therapy), confidence in study findings and measurement duration. It was observed that behavioural incentives (−0.49 SD, 95% CI [−0.85, −0.13]) have a more significant effect on problematic substance use outcomes than talking therapies (−0.26 SD, 95% CI [−0.37, −0.15]). Findings also indicate that short‐term impacts are greater on average than long‐term impacts (short‐term = −0.42 SD, 95% CI [−0.69, −0.14]; long‐term = −0.26 SD, 95% CI [−0.58, 0.06]). Finally, when we undertake a moderator analysis to explore the effect of low versus medium/high confidence studies, we observe slightly different average effect sizes compared to the combined average effect (low confidence = −0.39 SD, 95% CI [−0.58, −0.20]; medium/high confidence = −0.30 SD, 95% CI [−0.55, −0.06]).

To conclude, results show that psychosocial intervention are effective in terms of problematic substance use outcomes. They also indicate that intervention – on average – may be more effective short‐term.

#### 5.3.3 Reduction in mental ill health

**Figure 6:**
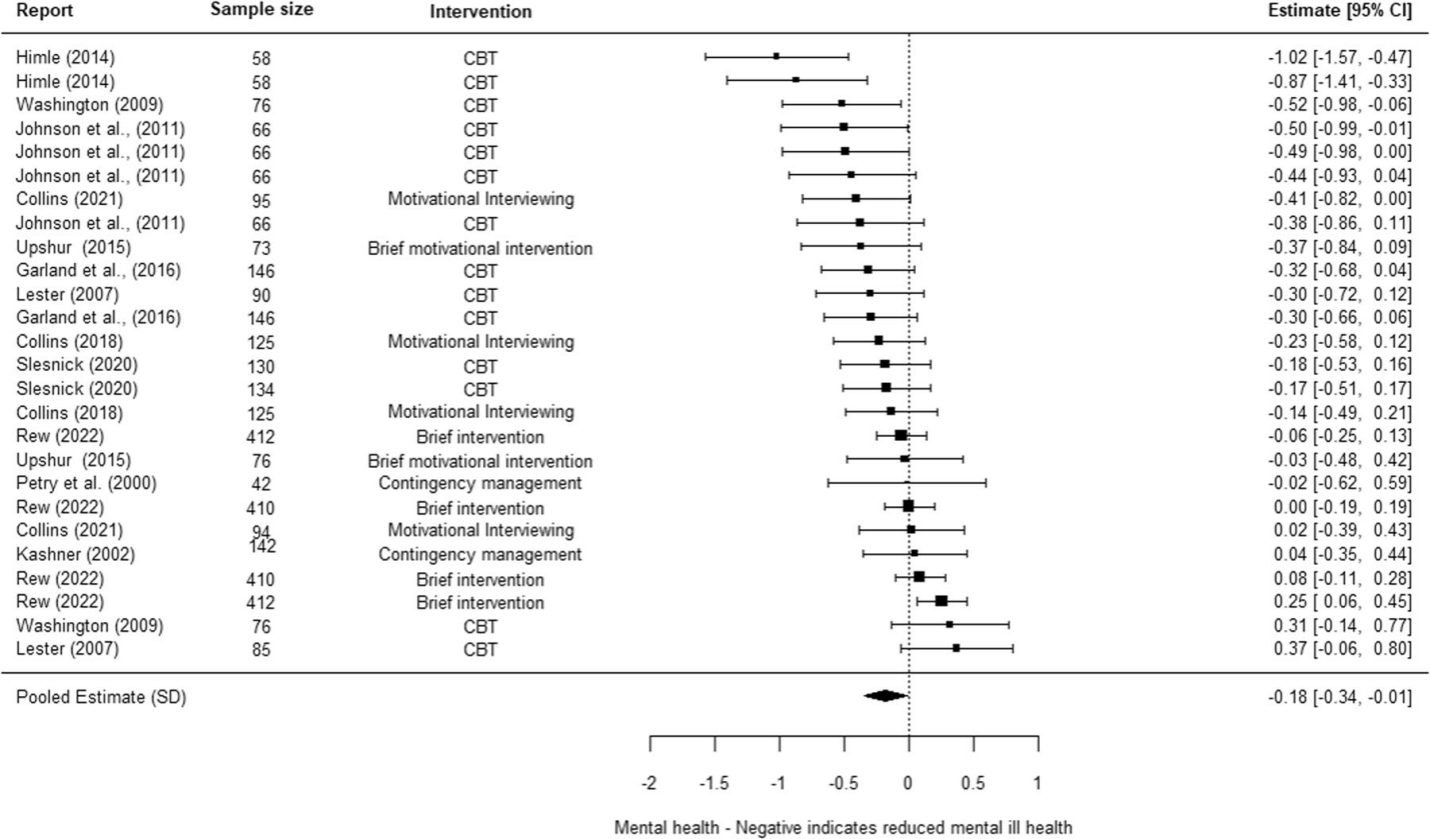
Effectiveness of psychosocial interventions on reducing mental ill health.

We were able to combine 26 effect sizes from 12 studies reported in 16 papers. The average effect of psychosocial interventions compared to TAU service provision in reducing mental ill health is (−0.18 SD, 95% CI [−0.34, −0.01]) with a prediction interval ranging between −0.70 and 0.35, which indicates a high level of heterogeneity (*I* ^2^ = 61.73%), suggesting that a random new psychosocial intervention could have a positive, null or negative effect on mental health. Figure 6 presents standardised effects by individual analysis points (no aggregation is applied).

Further sensitivity analysis indicates that results were sensitive to the removal of low confidence studies (−0.29 SD, 95% CI [−0.53, −0.05] – 7 studies, 15 effect sizes), suggesting an increase of the average effect once the lowest confidence studies are removed. Moreover, we conducted separate single variable meta‐regression models for two moderator variables – intervention intensity (high or low) and measurement duration – where at least 10 observations were available for each characteristic modelled. Findings indicate that interventions with high intensity are significantly more effective (−0.29 SD, 95% CI [−0.58, −0.01]) than low intensity interventions (−0.05 SD, 95% CI [−0.28, 0.18]). The other moderator variables (intervention type, demographics, study location, study design) were not powered due to the small number of studies available or were not statistically significant under conventional levels (*p* < 0.05). When we undertake a moderator analysis to explore the individual effects of short or long follow‐up points, we see the effect slightly diminishing long‐term (short‐term = 0.22 SD, 95% CI [−0.43, −0.01]; long‐term = −0.09 SD, 95% CI [−0.25, 0.07]). It should be noted that in several of these separate analyses, it is possible that the intervention tested is less effective than treatment as usual (i.e., the confidence intervals cross the line of no effect).

Overall, findings indicate that psychosocial interventions – on average – improve mental health outcomes (i.e., reduce mental ill health), but the confidence interval suggests that this average effect might be marginal.

#### 5.3.4 Reduction in housing instability

**Figure 7:**
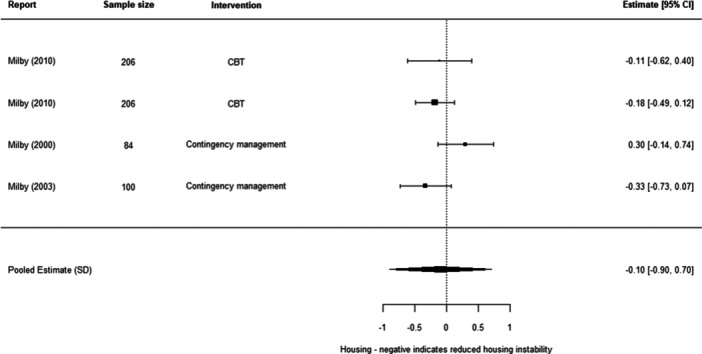
Effectiveness of psychosocial interventions of reducing housing instability.

We caution that the estimates for the outcomes related to housing instability are based on a small number of effect sizes and studies (i.e., 2 studies, 3 reports, 4 effect sizes), and the studies are from a single lead author. Drawing from the included studies, the average effect for psychosocial interventions in reducing housing instability is (−0.10 SD, 95% CI [−0.90, 0.70]) with a prediction interval ranging between −3.34 and 3.14 (*I* ^2^ = 59.29%), as illustrated in Figure 7.

Because we only identified two studies reporting this outcome, further statistical moderator, or sensitivity analyses are not possible for housing instability.

Whenever possible, we conducted Egger's tests for publication bias for each outcome. However, none of the tests were found to be statistically significant, therefore funnel plots are not included.

Conclusions on housing outcomes cannot be drawn from the presented results, given the high level of uncertainties around our estimates.

#### 5.3.5 RQ2. Are there differences in the effectiveness of psychosocial interventions in terms of their underlying theories of change?

Studies were reviewed to assess whether they provide any reference to the three meta‐theories of change (interpersonal relationships, habituation, and meta‐cognitive awareness) identified in the protocol for this review. There was no evidence, from any of the included studies, to suggest support for the review team's hypotheses about these three theories. Therefore, we did not undertake any statistical analysis relating to these theoretical domains.

Indeed, we generally found that these interventions were undertheorised in the included studies. Typically, the intervention design was justified by reference to existing studies, with little explanation of how the intervention is intended to lead to the expected outcomes. In several of the included studies, there was no discussion or references to how the intervention might work.

#### 5.3.6 RQ3. What is the effect of intervention types (e.g., talking therapies, behavioural incentives, self‐help) in terms of improving specific compared to treatment as usual?

Because of observed heterogeneity within each outcome cluster (each outcome type includes a variety of fundamentally different interventions, with varying levels of intensity), we have decided not to undertake meta‐analysis to answer research question 3. Instead, we only apply statistical tests to estimate the average effect of specific interventions and undertake narrative synthesis for each intervention when using meta‐analysis is not feasible (see research question 4).

#### 5.3.7 RQ4. What is the effect of individual interventions in terms of improving specific outcomes compared to treatment as usual?

We conducted meta‐analysis for each individual intervention against a unique outcome construct where at least two unique studies (with four or more effect sizes) are available. Table 2 presents the number of papers and related effect sizes by intervention and outcome (highlighted numbers indicate that interventions and related outcomes are adequate for meta‐analysis).

**Table 2:** Papers and effect sizes.

Intervention	Problematic substance use	Mental ill health	Housing instability
Brief Intervention	1 *(1)*	1 *(4)*	
Brief Motivational Intervention	**2** ***(4)***	1 *(2)*	
Cognitive Behavioural Therapy	2 *(2)*	**6** ***(14)***	1 *(2)*
Contingency Management	**6** ***(12)***	2 *(2)*	1 *(2)*
Dialectical Behavioural Therapy	1 *(2)*		
Motivational Interviewing	**3** ***(8)***	**2** ***(4)***	

##### CBT

###### Mental ill health outcomes

**Figure 8:**
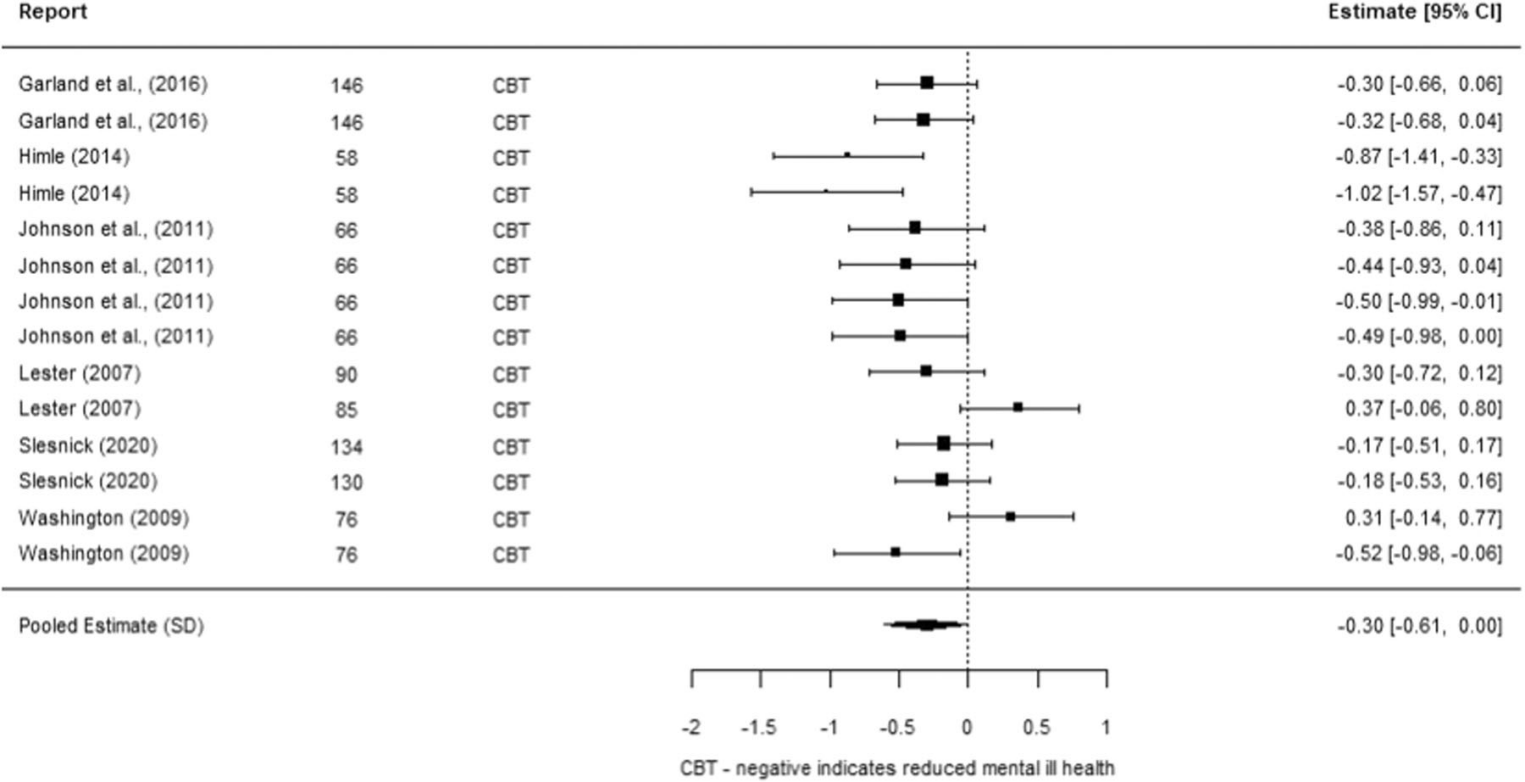
Effectiveness of cognitive behavioural therapy on reducing mental ill health.

Drawing from the included papers (6 papers, 14 effect sizes), the average effect for CBT in reducing mental ill health is (−0.30 SD, 95% CI [−0.61, 0.002]) with a prediction interval ranging from −1.09 to 0.48. The *I* ^2^ value also indicates high levels of heterogeneity (*I* ^2^ = 61.84%) (Figure 8).

No studies were classes as low confidence and no effect sizes were converted from odds ratios, therefore sensitivity tests were not carried out. Publication bias analysis is not possible due to low the number of papers.

###### Problematic substance use outcomes

Two studies examined the effect of CBT on this outcome.

Garland et al. (2017) evaluated two forms of CBT (standard and a revised version called Mindfulness‐Oriented Recovery Enhancement (MORE)) against treatment as usual, which the authors describe as a modified therapeutic community. A sample of *n* = 180 were randomly assigned to the two treatment groups (*n* = 64 to each) and TAU (*n* = 52). The authors examined a number of measures across several outcome domains, including problematic substance use. The authors report that MORE was more effective compared to TAU, with small but positive effects noted. The authors noted that they did not find the standard version of CBT to be effective. It is worth noting that the study only examined outcomes after 10 weeks of treatment, and the authors recognise that this is a limitation.

Milby et al. (2008) compared two treatments. Both interventions involve abstinent‐contingent housing, but the difference between them was a form of CBT called behavioural day treatment. A randomly selected sample size of *n* = 103 was assigned to each of the treatment groups. The authors examined measures of attendance and completion of treatment, as well as problematic substance use (urine‐test based measures of abstinence). The authors found a clear difference in abstinence between the two treatment groups, though conclude that: ‘the CM group achieved high and sustained abstinence throughout active treatment, which was not significantly different from, nor inferior to that of the more intense and expensive CM+ treatment’ (p. 10).

###### Housing instability outcomes

Drawing on the same study as Milby et al. (2008), Milby et al. (2010) found that there were no differences between the two treatment groups in relation to housing instability.

##### Contingency management

###### Problematic substance use outcomes

**Figure 9:**
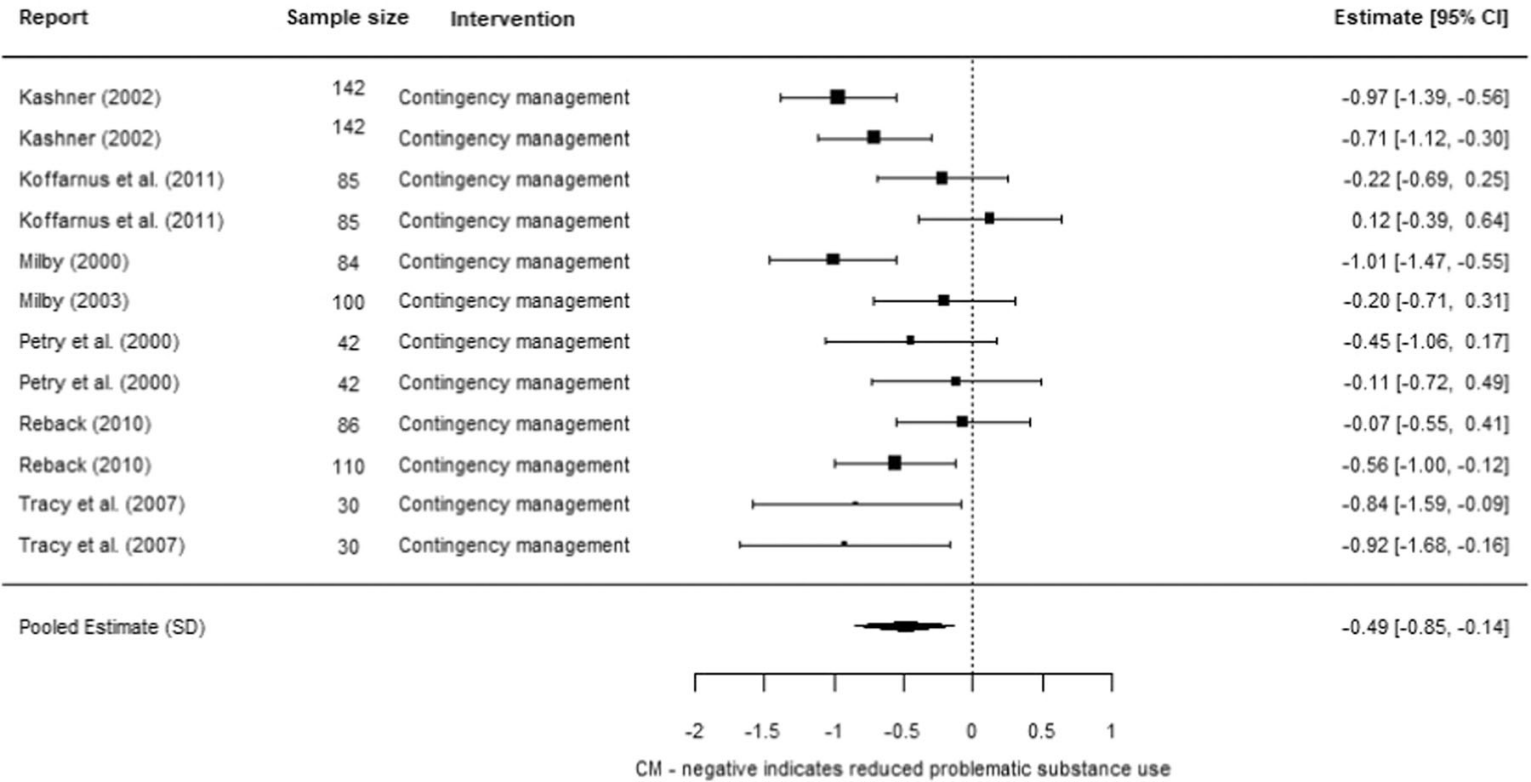
Effectiveness of contingency management on reducing problematic substance use.

Across the included studies (6 studies, 12 effect sizes), all but one of the reported results indicated an effect favouring the treatment group and one indicated an effect favouring the comparison. The weighted average favoured the treatment group and was statistically significant (−0.49 SD, 95% CI [−0.85, −0.14]) as evidenced in Figure 9, with a prediction interval ranging between −1.36 and 0.38. The *I* ^2^ value indicates that 58% of the total variance can be attributed to heterogeneity (*I* ^2^ = 58.03%).

These findings need to be treated with caution as four of the six included studies have been classed as low confidence. When low confidence studies are removed, the average treatment effect increases. The wide confidence interval indicates high levels of uncertainty (−0.59 SD, 95% CI [−3.83, 2.64]). Findings were also sensitive of the removal of effect sizes converted from odds ratios (−0.67 SD, 95% CI [−1.08, −0.25]). The number of studies did not allow us to conduct publication bias analysis.

###### Mental ill health outcomes

Two of our included studies examined the effect of contingency management on mental ill health. Kashner, (2002) evaluated a contingency based work programme for US veterans experiencing homelessness compared to a control group, where participants were provided with positive rewards for negative urine tests and adherence of outpatient addiction treatment appointments. Of *n* = 127 who started in the treatment group, *n* = 111 completed, with *n* = 35/31 respectively for the control group. The authors report no significant differences in the mental ill health outcomes between the treatment and control groups. Petry et al. (2000) also involves a contingency management intervention that uses positive rewards to incentivise abstinence. In this case, the authors evaluated standard treatment versus standard treatment with contingency management. A total of *n* = 42 participants were randomly assigned to either of these two treatments (*n* = 19 to standard treatment plus CM, *n* = 23 to standard treatment). The authors undertook several analyses, including a measure for ASI severity in mental ill health. Like the previous study discussed (Kashner, 2002), they found no significant difference between the control and treatment groups for the impact of contingency management on mental ill health outcomes.

###### Housing instability outcomes

None of the included studies examined the effect of contingency management on housing instability.

##### Motivational interviewing

###### Problematic substance use outcomes

**Figure 10:**
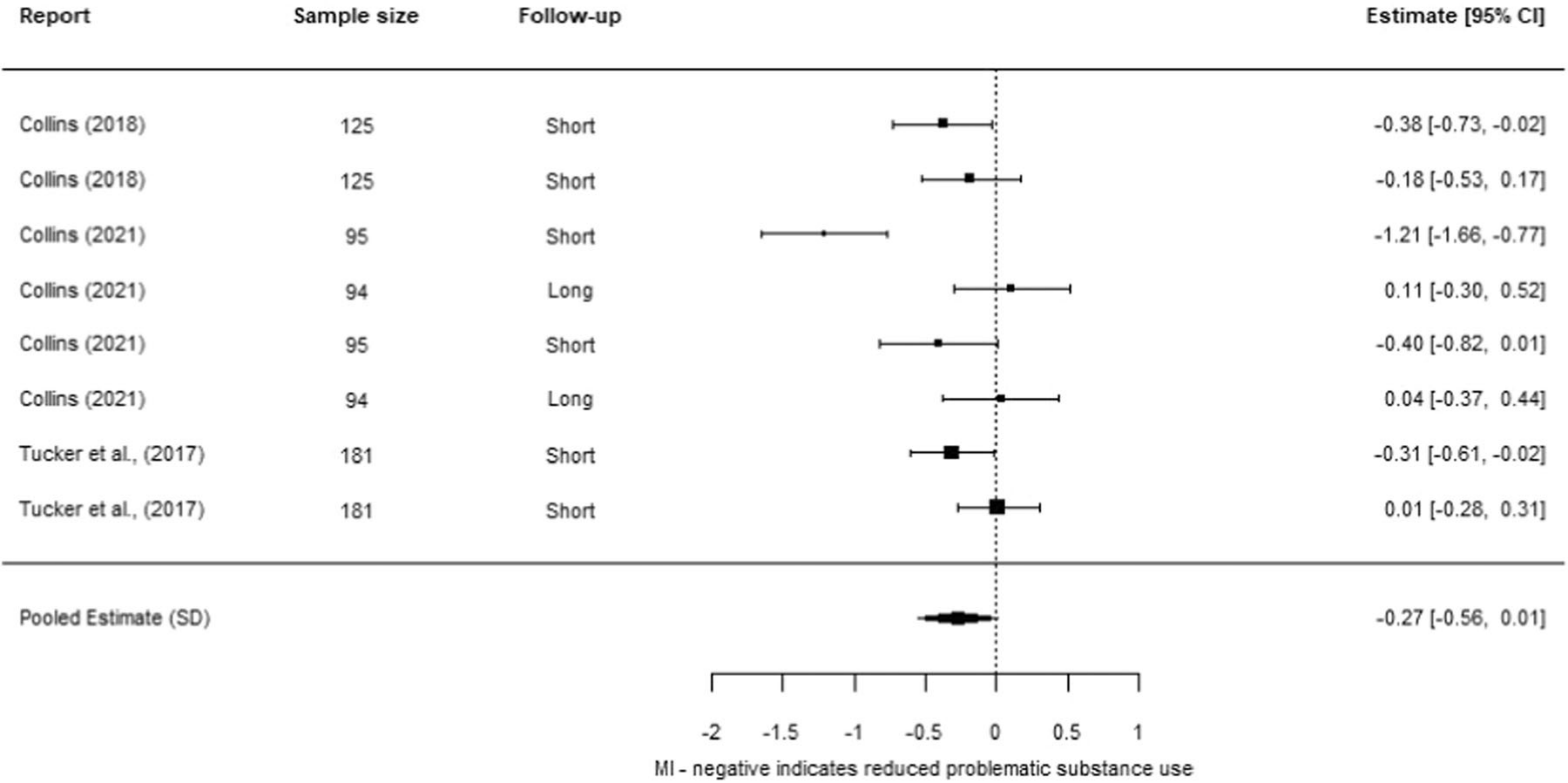
Effectiveness of motivational interviewing on reducing problematic substance use.

Drawing from three studies, and 14 effect sizes, the average effect of Motivational Interviewing in reducing substance use is (−0.27 SD, 95% [−0.56, 0.01]) as set out in Figure 10, with a wide prediction interval −2.07, 1.53 and high *I* ^2^ value (*I* ^2^ = 82.60) indicating high levels of heterogeneity.

Two of three included studies have been classed as low confidence. Sensitivity and publication bias analyses were not applicable given the low number of independent effect sizes.

###### Mental ill health outcomes

**Figure 11:**
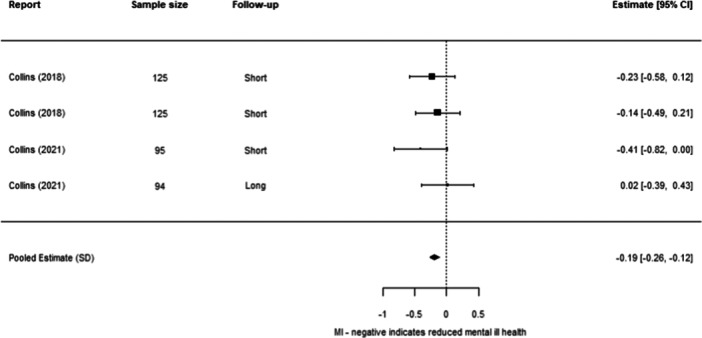
Effectiveness of motivational interviewing on reducing mental ill health.

The average effect of Motivational Interviewing was also assessed against mental ill health outcomes (2 papers, 4 effect sizes). Findings set out in Figure 11 indicate that – on average – Motivational Interviewing significantly improves mental health (−0.19 SD, 95% CI [−0.26, −0.12]) with a prediction interval ranging between 1.96 and 1.58, and an *I* ^2^ value of *I* ^2^ = 34.11.

Both included papers have been classed as low confidence. Sensitivity and publication bias analyses were not applicable given the low number of independent effect sizes.

###### Housing instability outcomes

None of the included papers examined the effect of Motivational Interviewing on housing instability.

##### BMI

###### Problematic substance use outcomes

**Figure 12:**
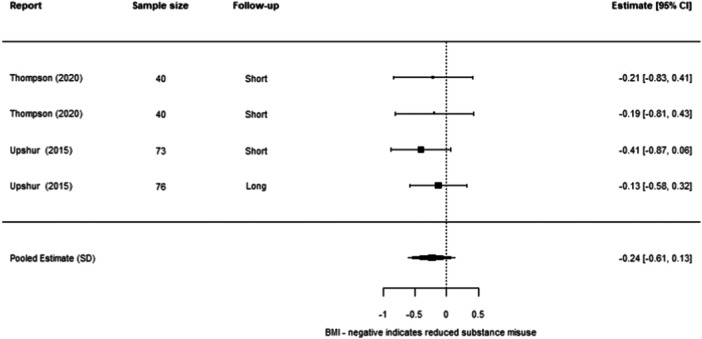
Effectiveness of brief motivational interventions on reducing problematic substance use.

As presented in Figure 12, two papers measured the effect of Brief Motivational Intervention against problematic substance use outcomes. Our results show a non‐significant average effect on reducing problematic substance use (−0.24 SD, 95% CI [−0.61, 0.13]), with a low amount of heterogeneity (*I* ^2^ = 1.20%) and a prediction interval ranging from −0.70 to 0.22.

Both included studies have been classed as high confidence. Publication bias analysis was not applicable given the low number of independent effect sizes.

###### Mental ill health outcomes

Upshur et al. (2015) was the only included paper to examine the effect of Brief Motivational Intervention on mental ill health. This study is unusual in that all participants were women. It compared an intervention called Project Renewal, a chronic care intervention that included several components, of which one was BMI. Eighty‐two women were randomly assigned to either the treatment group (*n* = 42) or treatment as usual (*n* = 40). The authors found no significant difference between the treatment and control groups in relation to mental ill health.

###### Housing instability outcomes

There were no studies that use outcomes measures related to housing instability.

##### BI

Two of the included studies examined the effectiveness of Brief Interventions, one on problematic substance use outcomes (Thompson et al., 2017) and mental ill health outcomes (Rew et al., 2022).

Thompson et al. (2017) compared a Brief Intervention treatment with an educational programme, targeted at young people experiencing homelessness and engaging in risky sexual behaviours and alcohol use. Participants were randomly assigned to the treatment (*n* = 30) or control (*n* = 31). The authors found that the intervention did not affect problematic substance use outcomes, either independently or compared to the educational programme.

Rew et al. (2022) also focused on young people experiencing homelessness, with an intervention aimed at reducing problematic substance use (both alcohol and drugs), and risky sexual behaviour. This RCT examined four conditions: (1) pre‐test and intervention; (2) pre‐test and control; (3) intervention only; and (4) control only. A total of *n* = 602 participants from Austin, Texas and Columbus, Ohio, were randomly assigned to one of these four groups, *n* = 150, *n* = 151, *n* = 151, and *n* = 150, respectively. The study examined ‘enhanced psychological capital’, using established measures of hope, optimism, resilience, self‐efficacy, and gratitude. The authors found statistically significant differences between treatment and control groups in several of these individual measures, although this effect disappeared at the end of the intervention.

##### DBT

One paper examined the effectiveness of this intervention and did so with respect to problematic substance use outcomes. Nyamathi et al. (2017) compared a modified version of this intervention with a health promotion control, in a study that examined women experiencing homelessness and having recently left prison. A total of *n* = 130 women were randomly assigned to either the treatment (*n* = 65) or control (*n* = 65) condition. The authors found that the intervention increased drug and alcohol abstinence at 6 month follow up, compared to the control.

#### 5.3.8 RQ5. Are there differences in the effectiveness of intervention types in terms of improving specific outcomes?

We intended to answer this question through pairwise comparison of intervention types using studies that made a direct comparison between specific psychosocial interventions. However, no studies were identified that compared such interventions. There were three studies that introduced slight modifications of the same psychosocial intervention to different trial arms. However, all of these were classified as alternate versions of the same intervention based on our classification, and therefore could not form the basis of pairwise comparison.

#### 5.3.9 RQ6. For whom do the interventions work best?

We intended to answer this question through meta‐analysis including all studies with group characteristic as a moderator (gender, race) or sub‐group analysis. However, we did not have enough effect sizes from different papers to conduct these analyses.

### 5.4 Summary of main results

Overall, we found that psychosocial interventions were more effective than treatment as usual for adults experiencing homelessness (overall effect size of −0.25 SD, 95% CI [−0.36, −0.13]). This analysis draws on 59 effect sizes from 23 papers, covering 20 separate studies. This analysis includes only 6 of the 20 interventions we identified as meeting our definition of psychosocial interventions and includes all 3 of our outcomes of interest (housing instability, problematic substance use and mental ill health). There is a significant level of heterogeneity across the studies included in this analysis, which is to be expected given the number of different interventions and outcome measures included, as well as the diversity of the studies' participants.

We also found that psychosocial interventions were more effective than treatment as usual in relation to all three of our individual outcomes of interest (although not significant in relation to housing instability). For problematic substance use, we found an average effect size of 9 (−0.34 SD, 95% CI [−0.48, −0.21]); for mental ill health of (−0.18 SD, 95% CI [−0.34, −0.01]); and for housing instability of (−0.10 SD, 95% [−0.90, 0.70]). These effect sizes are small but nevertheless encouraging; for both problematic substance use and mental ill health, the confidence intervals do not cross the line of no effect, suggesting these interventions work. The finding in relation to housing instability is less encouraging, and we should caution that it is based on three reports from two studies, all with the same lead author.

Our analysis of the effectiveness of individual psychosocial interventions is also encouraging. We were able to undertake meta‐analyses with respect to CBT, contingency management, motivational interviewing, and brief motivational interviewing. Table 3 summaries the results of the meta‐analyses, which are then described.

**Table 3:** Summary of findings.

Intervention	Problematic substance use	Mental ill health	Housing instability
Cognitive Behavioural Therapy		(−0.30 SD, 95% CI [−0.61, 0.002]	
Contingency Management	**(−0.49 SD, 95% CI [−0.85, −0.14])**		
Motivational Interviewing	(−0.27 SD, 95% CI [−0.56, 0.01])	**(−0.19 SD, 95% CI [−0.26, −0.12])**	
Brief Motivational Intervention	(−0.24 SD, 95% CI [−0.61, 0.13])		

*Note*: Significant findings are in bold.

### 5.5 Overall completeness and applicability of evidence

There is some good evidence of the effectiveness of individual psychosocial interventions for adults experiencing homelessness. There is more evidence in relation to some interventions, while others there is no effectiveness evidence at all. There is more evidence about the effectiveness of individual interventions in relation to reducing problematic substance use and reducing mental ill health, but is more limited in terms of reducing housing instability.

The 26 papers included in this review are all from the United States. Policy makers, funders, and service providers face significant challenges when trying to translate the evidence base in countries outside the United States, particularly where there are differences in the profile of adults experiencing homelessness, differences in access to health and social care services, and differences in homelessness services. In particular, the relatively better access to publicly funded healthcare services for people experiencing homelessness in the United Kingdom compared to the United States may reduce the difference between intervention of interest and treatment as usual if the intervention were implemented in the United Kingdom. (Though people experiencing homelessness in the United Kingdom are still less likely to be able to access primary and community health services, and be more dependent on emergency services, compared to the general population.)

Men are over‐represented in this evidence‐base, a finding that is consistent with other systematic reviews of the effectiveness evidence around homelessness (see, e.g., O'Leary et al. (2024)). Almost two‐thirds of the participants in the included papers are men, even though 4 of the included papers have participants who are solely women.

Finally, we found that the interventions were generally undertheorised in the included studies, with few explanations of how doing x might result in outcome y. While a small number of the studies did include details of the expected causal mechanisms through which interventions were expected to work, most relied on referencing previous studies, or did not provide any theoretical justification for the intervention's design. This is particularly significant given that many of the interventions included were multi‐component interventions, or were variations on previously evaluated interventions, and because people experiencing homelessness face a number of challenges over and above their problematic substance use.

### 5.6 Quality of the evidence

The evidence base suffers from a number of important methodological limitations. Although the evidence base is made up entirely of RCTs, many suffer from high rates of attrition, including differential attrition. While this is unsurprising for this population, it can both reduce the sample size of a study, making it more difficult to detect a difference between groups, as well as introduce bias when there is differential attrition between the intervention and comparison groups. In addition, the vast majority of papers did not report that they used masking/blinding of outcome assessors or masking/blinding of the team to the intervention allocation during analysis. This is important where there is an element of judgement on the part of the assessor during the collection of outcome data or in the analysis of data, as they may behave in ways that differentially affect the outcomes in different treatment groups. Few studies reported that they used power calculations to determine their sample size, and many of the studies had small sample sizes. This limits the ability of the studies to detect a difference between groups, particularly if the expected effect size for this sort of intervention is small. Finally, many of the studies did not provide detailed explanations of the treatment as usual services to which their intervention of interest was being compared. Not all of the studies compared to treatment as usual, with some comparing an intervention of interest with variations of that intervention.

### 5.7 Potential biases in the review process

The main source of papers (*n* = 18/26) for inclusion in title and abstract reviews were searches undertaken by the Campbell Collaboration for the 5th edition of the Homelessness Effectiveness Evidence and Gap Map (Jain et al., 2023), and as such, the review team did not undertake these searches. There is a significant degree of heterogeneity of studies included in the meta‐analyses, particularly those analyses that address the first research question (the effectiveness of psychosocial interventions overall, and the effectiveness of psychosocial interventions over for each of the three outcomes of interest). This in part reflects the broad scope of this review, and the significant differences between interventiONS that fall under the banner of being ‘psychosocial’, It also possibly reflects methodological limitations of the included studies. Because of an insufficient number of studies and effect sizes, we were not able to include all the moderating variables in one analysis, and therefore had to undertake single characteristic meta‐regression where possible. Any conclusions drawn from meta‐regression analysis should always be cautious and exploratory given that these relationships are typically observational in nature and based on a small number of effects. With single characteristic meta‐regression, the identified moderators may be related to one another and the analysis can therefore be misleading.

### 5.8 Agreements and disagreements with other studies or reviews

There are no other published systematic reviews that examine the effectiveness of psychosocial interventions for this population that are directly comparable to the review set out here. There are two reviews that complement ours: one, by Scott and colleagues (pending) is currently under review. It is not registered with the Campbell Collaboration and focuses on psychosocial outcomes rather than psychosocial interventions. The second is by Hyun et al. (2020). This review focused on the psychosocial outcomes of psychosocial interventions for adults experiencing homelessness. Through meta‐analysis, the Hyun review (2020) considered outcomes in relation to depression, anxiety, mental health status, PTSD symptoms, psychological distress, self‐efficacy, and quality of life, and did so in relation to two interventions, namely case management and CBT. The reviewers also examined other psychosocial interventions narratively. Hyun et al. (2020) found mixed evidence of the effectiveness of psychosocial interventions. They found psychosocial interventions to be effective in reducing anxiety and enhancing mental health status, and some evidence that CBT might be effective. They also were unable to compare the effectiveness of different types of psychosocial interventions. These findings generally complement those outlined in our review.

## 6 DISCUSSION

### 6.1 Summary of main results

Overall, we found that psychosocial interventions were more effective than treatment as usual for adults experiencing homelessness (overall effect size of −0.25 SD, 95% CI [−0.36, −0.13]). This analysis draws on 59 effect sizes from 23 papers, covering 20 separate studies. This analysis includes only 6 of the 20 interventions we identified as meeting our definition and typology of psychosocial interventions, and includes all three of our outcomes of interest (housing instability, problematic substance use and mental ill health). There is a significant level of heterogeneity across the studies included in this analysis, which is to be expected given the number of different interventions and outcome measures included, as well as the diversity of the studies' participants.

We also found that psychosocial interventions were more effective than treatment as usual/comparator intervention in relation to all three of our individual outcomes of interest (although not significant in relation to housing instability). For substance use, we found an average effect size of (−0.34 SD, 95% CI, [−0.48, −0.21]); for mental ill health of (−0.18 SD, 95% CI [−0.34, −0.01]); and for housing instability of (−0.10 SD, 95% CI [−0.90, 0.70]). These effect sizes are small but nevertheless encouraging; for both substance use and mental ill health, the confidence intervals do not cross the line of no effect, suggesting these interventions work. The finding in relation to housing instability is less encouraging, and we should caution that it is based on three reports from two studies, all with the same lead author.

Our analysis of the effectiveness of individual psychosocial interventions is also encouraging. We were able to undertake meta analyses with respect to CBT, contingency management, motivational interviewing, and brief motivational interviewing.

In relation to CBT, we found that this intervention led to reduced mental ill health, although the finding is not significant (−0.30 SD, 95% CI [−0.61, 0.002]), drawing on 14 effect sizes from 6 studies. We were unable to complete meta‐analysis with respect to substance use or housing stability. Two studies examined the effect of CBT on substance use (Garland et al., 2017; Milby et al., 2008), and one study, Milby et al. (2010), examined this intervention's effect on housing instability.

Contingency management was found to be effective (although not significant) in reducing problematic substance use (−0.49 SD, 95% CI [−0.85, −0.14]), based on 12 effect sizes in 6 of the included studies. Two studies – Kashner (2002) and Petry et al. (2000) – examined the effect on mental ill health, and there were no studies that measured housing instability.

Motivational Interviewing was found to be effective in reducing problematic substance use (−0.27 SD, (95% CI [−0.56, 0.01]) (not significant) and mental ill health (−0.19 SD, 95% CI [−0.26, −0.12]) (not significant), based on 3 studies and 14 effect sizes and 2 studies and 4 effect sizes, respectively. No studies used measures of housing instability.

Finally, Brief Motivational Interventions were found to be effective in reducing problematic substance use (−0.24 SD, 95% CI [−0.61, 0.13]) (not significant). One paper, Upshur et al. (2015), examined their effectiveness in relation to mental ill health, and there were no studies that looked at housing instability.

We were able to undertake narrative synthesis in relation to Brief Interventions and Dialectic Behavioural Therapy. There were a number of psychosocial interventions of interest to us and set out in our typology in Table 1 for which we were unable to locate any effectiveness studies. These include skills training; 12‐step facilitation therapy; cue exposure treatment; non‐contingent rewards; motivational enhancement therapy; family therapy/couples therapy/community reinforcement; therapeutic communities/residential rehabilitation; social behaviour and network therapy; relapse prevention; mentalisation based therapy; 12‐step programmes, and SMART.

## 7 AUTHORS' CONCLUSIONS

### 7.1 Implications for practice and policy

This systematic review sought to understand the effectiveness of psychosocial interventions for adults experiencing homelessness, in relation to reducing problematic substance use, reducing mental ill health, and reducing housing instability. The review points to the potential benefits of this group of interventions, with some encouraging results in relation to some specific interventions and outcomes. It is worth stressing that, where we were able to calculate effect sizes, these were often small and, in many cases, crossed the line of no effect (i.e., there is a chance that they are less effective than treatment as usual). Significant heterogeneity between studies and high rates of attrition in many studies make it difficult to have high confidence in the interventions.

We suggest two implications for practice and policy arising from this review.

#### 7.1.1 Gaps in the evidence

While there is evidence to suggest that psychosocial interventions might be effective for this population, some of this evidence is mixed, and there are gaps that need to be taken into account.

Each of the 26 studies included in this review is from the United States. There are significant differences in the profile of people experiencing homelessness and in the provision of publicly funded services (particularly healthcare) between the United States and other developed countries. As such, it is not possible to simply translate evidence of effectiveness in the United States and assume these interventions will also be effective elsewhere.

The effectiveness evidence is concentrated on a small number of individual and generally well‐established psychosocial interventions, with a greater focus on substance use and mental ill health outcomes than on housing outcomes. This does not mean that other psychosocial interventions are not effective. However, the lack of a single definition of what constitutes psychosocial interventions and the lack of a single, coherent underlying theory of how these interventions might affect change means that we caution against an assumption that evidence of effectiveness for one type of psychosocial intervention could be understood as evidence of effectiveness of all types of these interventions.

#### 7.1.2 Motivation could be important

Of the four individual interventions for which we were able to undertake meta‐analysis, three arguably focus on the motivation of service users to achieve change, namely contingency management, motivational interviewing, and brief motivational interventions. Previous studies have also found contingency management to be effective (see, e.g., O'Leary et al., 2024), though there are questions as to whether these interventions lead to lasting changes. Our review of the qualitive evidence of the experiences of adults experiencing homelessness when they access or use psychosocial interventions identifies the key role that the individual service users plays in their own change journey, and that an individual's motivation, sense of hope, and the goals they set are important ingredients for making change happen. We recognise that motivation is a complex and contested term, and that this review did not directly look at motivation as a causal ingredient. We therefore argue that further thought and research needs to be given to the role that motivation may play in the success of psychosocial interventions for this population.

### 7.2 Implications for research

We found that many of the studies were of low methodological quality, and the interventions of interest were generally under‐theorised. There are obvious challenges with undertaken research in this field, but more needs to be done to improve the methodological rigour of research in this area.

Secondly, the evidence base is entirely from the United States. The lack of effectiveness studies from the United Kingdom is disappointing, particularly given the focus of governments over the last 25 years on tackling homelessness. More research is needed in the United Kingdom that would allow policy makers and practitioners to focus on interventions that are effective.

There is also a clear gender bias in the underlying research. While it is the case that men are more likely to experience the more visible and extreme forms of homelessness, and are also more likely to enter treatment for problematic substance use, we would still argue that more research is needed about the experiences of, and what interventions are effective for, women experiencing homelessness. Finally, some of the language used in the included studies can be dehumanising. Given the significant barriers that some who are experiencing homelessness can face when accessing public services, it is important that researchers do not add to or reinforce these barriers by using dehumanising language.

## CONTRIBUTIONS OF AUTHORS

- Content: Dr. Chris O'Leary.
- Systematic review methods: Dr. Esther Coren.
- Statistical analysis: Sandor Gellen.
- Information retrieval: Harry Armitage, Anton Roberts.

## DECLARATIONS OF INTEREST

None to declare.

## PLANS FOR UPDATING THIS REVIEW

Plans to update this review are subject to further funding.

## DIFFERENCES BETWEEN PROTOCOL AND REVIEW

None to report.

## SOURCES OF SUPPORT

### Internal sources

#### External sources

National Institute for Health Research, UK. Evidence synthesis grant no. 133287.

## Supporting information

Supporting information.

Supporting information.

Supporting information.
