# Supplementary material for: The effectiveness of psychosocial interventions for reducing problematic substance use, mental ill health, and housing instability in people experiencing homelessness in high income countries: A systematic review and meta‐analysis
Source: Campbell Syst Rev. 2025 Jan 17;21(1):e70019. doi: 10.1002/cl2.70019 (PMC11739802; doi:10.1002/cl2.70019)
Supplement: Supplementary file 2 — Supporting information. [file CL2-21-e70019-s002.docx]

Appendix – excluded studies with reason for exclusion

| **Study** | **Reason for exclusion** |
| --- | --- |
| Alessi and Petry (2013) | Not a population of interest. |
| Beattie et al. (2019) | No comparison group. |
| Clifasefi et al. (2020) | Not a relevant intervention. |
| Cox and Stoltenberg (1991) | Not a population of interest. |
| Fisk et al. (2006) | No comparison group. |
| Fletcher and Reback (2013) | Secondary analysis of data from included study. Duplicate. |
| Fors and Jarvis (1995) | Not a population of interest. (Did not meet 40 percent adults experiencing homelessness threshold.) |
| Frisman et al. (2009) | Comparison/design. |
| Glendening et al. (2020) | Not a population of interest. |
| Graham-Jones et al. (2004) | Not a relevant intervention. |
| Hyun et al. (2005) | Not a population of interest. (Did not meet 40 percent adults experiencing homelessness threshold.) |
| Kadoura (2014) | Not a population of interest. (Homeless families.) |
| Kennedy et al. (2022) | Not a population of interest. |
| Koffarnus et al. (2018) | Secondary analysis of data from included study. Duplicate. |
| Krabbenborg et al. (2015) | Cannot locate paper. |
| Krabbenborg et al. (2017) | Not a population of interest. |
| LePage and Garcia-Rea (2012) | Comparison/design. |
| LePage et al. (2006) | Unable to extract data. |
| Linn et al. (2003) | Unable to extract data. |
| McCay E et al. (2011) | Comparison/design. |
| Medalia et al. (2017) | Comparison/design. |
| Milby et al. (2005) | Duplicate. |
| Morris and Warnock (2001) | Unable to extract data. |
| Nolan (2006) | Extracted. |
| Nyamathi et al. (2016) | Comparison/design. |
| O'Campo et al. (2016) | Not an intervention of interest. |
| Ogden (2013) | Unable to extract data. |
| Peterson et al. (2006) | Not a population of interest. |
| Proehl (2007) | Comparison/design. |
| Rash et al. (2017) | Comparison/design. |
| Reilly et al. (2004) | Not an intervention of interest. |
| Ricelli (2019) | Unable to extract data. |
| Rodriguez-Moreno et al. (2022) | Comparison/design. |
| Rotheram-Borus et al. (2003) | Not a population of interest. |
| Sacks et al. (2004) | Not a population of interest. |
| Santa Maria (2019) | Comparison/design. |
| Schumacher et al. (2002) | Extracted. |
| Schumacher et al. (2003) | Secondary analysis of data from included study. Duplicate. |
| Shaner et al. (1997) | Comparison/design. |
| Shern et al. (2000) | Not an intervention of interest. |
| Slesnick et al (2005) | Duplicate. |
| Slesnick and Prestopnik (2005) | Not a population of interest. |
| Slesnick et al. (2007) | Not a population of interest. |
| Slesnick and Kang (2008) | Not a population of interest. |
| Slesnick and Prestopnik (2009) | Not a population of interest. |
| Slesnick et al. (2015) | Not a population of interest. |
| Slesnick et al. (2016) | Not a population of interest. |
| Smelson et al. (2018) | Not an intervention of interest. |
| Smith et al. (1998) | Comparison/design. |
| Stahler et al. (1995) | Unable to extract data. |
| Stahler et al. (2005) | Not a population of interest. |
| Theodos et al. (2016) | Not a population of interest. |
| van Kranenburg et al. (2019) | Comparison/design. |
| Wong et al. (2005) | Unable to locate paper. |
| Wu et al. (2022) | Secondary analysis of data from included study. Duplicate. |
| Zhang and Slesnick (2018) | Not a population of interest. (Did not meet 40 percent adults experiencing homelessness threshold.) |
| Zhang et al. (2018) | Not an intervention of interest. |

Alessi, S. M., & Petry, N. M. (2013). A randomized study of cellphone technology to reinforce alcohol abstinence in the natural environment. *Addiction*, *108*(5), 900-909. <https://doi.org/10.1111/add.12093>

Beattie, K., McCay, E., Aiello, A., Howes, C., Donald, F., Hughes, J., MacLaurin, B., & Organ, H. (2019). Who benefits most? A preliminary secondary analysis of stages of change among street-involved youth. *Archives of Psychiatric Nursing*, *33*(2), 143-148. <https://doi.org/10.1016/j.apnu.2018.11.011>

Clifasefi, S. L., Collins, S. E., & Board, L. A. (2020). The life-enhancing alcohol-management program: Results from a 6-month nonrandomized controlled pilot study assessing a community based participatory research program in housing first. *Journal of Community Psychology*, *48*(3), 763-776. <https://doi.org/10.1002/jcop.22291>

Cox, J. W., & Stoltenberg, C. D. (1991). Evaluation of a Treatment Program for Battered Wives. *Journal of Family Violence*, *6*(4), 395-413. <https://doi.org/Doi> 10.1007/Bf00980541

Fisk, D., Rakfeldt, J., & McCormack, E. (2006). Assertive outreach: An effective strategy for engaging homeless persons with substance use disorders into treatment. *American Journal of Drug and Alcohol Abuse*, *32*(3), 479-486. <https://doi.org/10.1080/00952990600754006>

Fletcher, J. B., & Reback, C. J. (2013). Antisocial personality disorder predicts methamphetamine treatment outcomes in homeless, substance-dependent men who have sex with men. *Journal of Substance Abuse Treatment*, *45*(3), 266-272. <https://doi.org/10.1016/j.jsat.2013.03.002>

Fors, S. W., & Jarvis, S. (1995). Evaluation of a peer-led drug abuse risk reduction project for runaway homeless youths. *Journal of Drug Education*, *25*(4), 321-333. <https://doi.org/Doi> 10.2190/Tu92-Lx8w-G7fd-9lem

Frisman, L. K., Mueser, K. T., Covell, N. H., Lin, H. J., Crocker, A., Drake, R. E., & Essock, S. M. (2009). Use of Integrated Dual Disorder Treatment Via Assertive Community Treatment Versus Clinical Case Management for Persons With Co-Occurring Disorders and Antisocial Personality Disorder. *Journal of Nervous and Mental Disease*, *197*(11), 822-828. <https://doi.org/10.1097/NMD.0b013e3181beac52>

Glendening, Z. S., Shinn, M., Brown, S. R., Cleveland, K. C., Cunningham, M. K., & Pergamit, M. R. (2020). Supportive housing for precariously housed families in the child welfare system: Who benefits most? *Children and Youth Services Review*, *116*. <https://doi.org/ARTN> 105206

10.1016/j.childyouth.2020.105206

Graham-Jones, S., Reilly, S., & Gaulton, E. (2004). Tackling the needs of the homeless: a controlled trial of health advocacy. *Health & Social Care in the Community*, *12*(3), 221-232. <https://doi.org/DOI> 10.1111/j.1365-2524.2004.00491.x

Hyun, M. S., Chung, H. I. C., & Lee, Y. J. (2005). The effect of cognitive-behavioral group therapy on the self-esteem, depression, and self-efficacy of runaway adolescents in a shelter in South Korea. *Applied Nursing Research*, *18*(3), 160-166. <https://doi.org/10.1016/j.apnr.2004.07.006>

Kadoura, W. E. (2014). *The brighter future for homeless families and their preschoolers' program in Salt Lake City, Utah* University of Utah].

Kennedy, D. P., Osilla, K. C., Hunter, S. B., Golinelli, D., Hernandez, E. M., & Tucker, J. S. (2022). Restructuring personal networks with a Motivational Interviewing social network intervention to assist the transition out of homelessness: A randomized control pilot study. *Plos One*, *17*(1). <https://doi.org/ARTN> e0262210

10.1371/journal.pone.0262210

Koffarnus, M. N., Bickel, W. K., & Kablinger, A. S. (2018). Remote Alcohol Monitoring to Facilitate Incentive-Based Treatment for Alcohol Use Disorder: A Randomized Trial. *Alcohol-Clinical and Experimental Research*, *42*(12), 2423-2431. <https://doi.org/10.1111/acer.13891>

Krabbenborg, M. A. M., Boersma, S. N., Beijersbergen, M. D., Goscha, R. J., & Wolf, J. R. L. M. (2015). Fidelity of a Strengths-Based Intervention Used by Dutch Shelters for Homeless Young Adults. *Psychiatric Services*, *66*(5), 470-476. <https://doi.org/10.1176/appi.ps.201300425>

Krabbenborg, M. A. M., Boersma, S. N., van der Veld, W. M., van Hulst, B., Vollebergh, W. A. M., & Wolf, J. R. L. M. (2017). A Cluster Randomized Controlled Trial Testing the Effectiveness of Houvast: A Strengths-Based Intervention for Homeless Young Adults. *Research on Social Work Practice*, *27*(6), 639-652. <https://doi.org/10.1177/1049731515622263>

LePage, J. P., Bluitt, M., McAdams, H., Merrell, C., House-Hatfield, T., & Garcia-Rea, E. (2006). Effects of increased social support and lifestyle behaviors in a domiciliary for homeless veterans. *Psychological Services*, *3*(1), 16-24.

LePage, J. P., & Garcia-Rea, E. A. (2012). Lifestyle Coaching's Effect on 6-Month Follow-Up in Recently Homeless Substance Dependent Veterans: A Randomized Study. *Psychiatric Rehabilitation Journal*, *35*(5), 396-402. <https://doi.org/10.1037/h0094500>

Linn, J. G., Neff, J. A., Theriot, R., Harris, J. L., & Graham, M. E. (2003). Reaching impaired populations with HIV prevention programs: A clinical trial for homeless mentally ill African-American men. *Cellular and Molecular Biology*, *49*(7), 1167-1175. <Go to ISI>://WOS:000187582700022

McCay E, Quesnel S, Langley J, Beanlands H, Cooper L, Blidner R, Aiello A, Mudachi N, Howes C, & A, B. K. (2011). A relationship-based intervention to improve social connectedness in street-involved youth: a pilot study. *Journal of Child and Adolescent Psychiactric Nursing*, *24*(4), 2018-2015.

Medalia, A., Saperstein, A. M., Huang, Y. L., Lee, S., & Ronan, E. J. (2017). Cognitive Skills Training for Homeless Transition-Age Youth: Feasibility and Pilot Efficacy of a Community Based Randomized Controlled Trial. *Journal of Nervous and Mental Disease*, *205*(11), 859-866. <https://doi.org/10.1097/Nmd.0000000000000741>

Milby, J. B., Schumacher, J. E., Wallace, D., Freedman, M. J., & Vuchinich, R. E. (2005). To house or not to house: The effects of providing housing to homeless substance abusers in treatment. *American Journal of Public Health*, *95*(7), 1259-1265. <https://doi.org/10.2105/Ajph.2004.039743>

Morris, D., & Warnock, J. (2001). Effectiveness of a Mobile Outreach and Crisis Services unit in reducing psychiatric symptoms in a population of homeless persons with severe mental illness. *The Journal of the Oklahoma State Medical Association*, *94*(8), 343-346.

Nolan, T. C. (2006). Outcomes for a transitional living program serving LGBTQ youth in New York City. *Child Welfare*, *85*(2), 385-406. <Go to ISI>://WOS:000238594900016

Nyamathi, A. M., Reback, C. J., Shoptaw, S., Salem, B. E., Zhang, S., Farabee, D., & Khalilifard, F. (2016). Impact of Community-Based Programs on Incarceration Outcomes Among Gay and Bisexual Stimulant-Using Homeless Adults. *Community Mental Health Journal*, *52*(8), 1037-1042. <https://doi.org/10.1007/s10597-014-9792-x>

O'Campo, P., Stergiopoulos, V., Nir, P., Levy, M., Misir, V., Chum, A., Arbach, B., Nisenbaum, R., To, M. J., & Hwang, S. W. (2016). How did a Housing First intervention improve health and social outcomes among homeless adults with mental illness in Toronto? Two-year outcomes from a randomised trial. *Bmj Open*, *6*(9). <https://doi.org/ARTN> e010581

10.1136/bmjopen-2015-010581

Ogden, S. M. (2013). *“IT FEELS LIKE HOME”: THE IMPACTS OF SUPPORTIVE HOUSING ON MALE YOUTH–PERSPECTIVES OF YOUTH AND SERVICE PROVIDERS AT FIVE BEDS TO HOME..*

Peterson, P. L., Baer, J. S., Wells, E. A., Ginzler, J. A., & Garrett, S. B. (2006). Short-term effects of a brief motivational intervention to reduce alcohol and drug risk among homeless adolescents. *Psychology of Addictive Behaviors*, *20*(3), 254-264. <https://doi.org/10.1037/0893-164x.20.3.254>

Proehl, R. A. (2007). Social justice, respect, and meaning-making: Keys to working with the homeless elderly population. *Health & Social Work*, *32*(4), 301-307. <https://doi.org/DOI> 10.1093/hsw/32.4.301

Rash, C. J., Alessi, S. M., & Petry, N. M. (2017). Substance Abuse Treatment Patients in Housing Programs Respond to Contingency Management Interventions. *Journal of Substance Abuse Treatment*, *72*, 97-102. <https://doi.org/10.1016/j.jsat.2016.07.001>

Reilly, S., Graham-Jones, S., Gaulton, E., & Davidson, E. (2004). Can a health advocate for homeless families reduce workload for the primary healthcare team? A controlled trial. *Health & Social Care in the Community*, *12*(1), 63-74. <https://doi.org/DOI> 10.1111/j.1365-2524.2004.00469.x

Ricelli, S. E. (2019). *Problem-Solving Therapy to Foster Resilience among Veterans Who Are Homeless or At-Risk for Homelessness: A Pilot Randomized Controlled*

*Trial* Drexel University].

Rodriguez-Moreno, S., Farchione, T. J., Roca, P., Marín, C., Guillén, A. I., & Panadero, S. (2022). Initial Effectiveness Evaluation of the Unified Protocol for Transdiagnostic Treatment of Emotional Disorders for Homeless Women. *Behavior Modification*, *46*(3), 506-528. <https://doi.org/Artn> 0145445520982562

10.1177/0145445520982562

Rotheram-Borus, M., Song, J., Gwadz, M., Lee, M., Van Rossem, R., & Koopman, C. (2003). Reductions in HIV Risk Among Runaway Youth. *Prevention Science*, *4*(3), 173-187.

Sacks, S., Sacks, J. Y., McKendrick, K., Pearson, F. S., Banks, S., & Harle, M. (2004). Outcomes from a therapeutic community for homeless addicted mothers and their children. *Administration and Policy in Mental Health and Mental Health Services Research*, *31*(4), 313-338. <Go to ISI>://WOS:000222327800002

Santa Maria, D. (2019). Development and Piloting of a Just-in-Time, Personalized HIV Prevention Intervention for Youth Experiencing Homelessness and Unstable Housing. In: clinicaltrials.gov.

Schumacher, J., Mennemeyer ST, Milby JB, Wallace D, & K., N. (2002). Costs and effectiveness of substance abuse treatments for homeless persons. *Journal of Health Policy Economics*, *5*(1), 33-42.

Schumacher, J. E., Milby, J. B., Wallace, D., Simpson, C., Frison, S., McNamara, C., & Usdan, S. (2003). Diagnostic compared with abstinence outcomes of day treatment and contingency management among cocaine-dependent homeless persons. *Experimental and Clinical Psychopharmacology*, *11*(2), 146-157. <https://doi.org/10.1037/1064-1297.11.2.146>

Shaner, A., Roberts, L. J., Eckman, T. A., Tucker, D. E., Tsuang, J. W., Wilkins, J. N., & Mintz, J. (1997). Monetary reinforcement of abstinence from cocaine among mentally ill patients with cocaine dependence. *Psychiatric Services*, *48*(6), 807-810. <Go to ISI>://WOS:A1997XB45200011

Shern, D. L., Tsemberis, S., Anthony, W., Lovell, A. M., Richmond, L., Felton, C. J., Winarski, J., & Cohen, M. (2000). Serving street-dwelling individuals with psychiatric disabilities: Outcomes of a psychiatric rehabilitation clinical trial. *American Journal of Public Health*, *90*(12), 1873-1878. <https://doi.org/Doi> 10.2105/Ajph.90.12.1873

Slesnick, N., Feng, X., Guo, X. M., Brakenhoff, B., Carmona, J., Murnan, A., Cash, S., & McRee, A. L. (2016). A Test of Outreach and Drop-in Linkage Versus Shelter Linkage for Connecting Homeless Youth to Services. *Prevention Science*, *17*(4), 450-460. <https://doi.org/10.1007/s11121-015-0630-3>

Slesnick, N., Guo, X. M., Brakenhoff, B., & Bantchevska, D. (2015). A Comparison of Three Interventions for Homeless Youth Evidencing Substance Use Disorders: Results of a Randomized Clinical Trial. *Journal of Substance Abuse Treatment*, *54*, 1-13. <https://doi.org/10.1016/j.jsat.2015.02.001>

Slesnick, N., & Kang, M. J. (2008). The impact of an integrated treatment on HIV risk behavior among homeless youth: a randomized controlled trial. *Journal of Behavioral Medicine*, *31*(1), 45-59. <https://doi.org/10.1007/s10865-007-9132-5>

Slesnick, N., Prestopnik, J., & Meyers, R. (2005). *Outcome of CRA with Homeless Adolescents: Preliminary findings* 67th Annual Scientific Meeting of the College on Problems of Drug Dependence, Orlando, Florida. <https://www.cochranelibrary.com/central/doi/10.1002/central/CN-00591039/full>

Slesnick, N., & Prestopnik, J. L. (2005). Ecologically based family therapy outcome with substance abusing runaway adolescents. *Journal of Adolescence*, *28*(2), 277-298. <https://doi.org/10.1016/j.adolescence.2005.02.008>

Slesnick, N., & Prestopnik, J. L. (2009). Comparison of Family Therapy Outcome With Alcohol-Abusing, Runaway Adolescents. *Journal of Marital and Family Therapy*, *35*(3), 255-277. <https://doi.org/10.1111/j.1752-0606.2009.00121.x>

Slesnick, N., Prestopnik, J. L., Meyers, R. J., & Glassman, M. (2007). Treatment outcome for street-living, homeless youth. *Addictive Behaviors*, *32*(6), 1237-1251. <https://doi.org/10.1016/j.addbeh.2006.08.010>

Smelson, D. A., Chinman, M., Hannah, G., Byrne, T., & McCarthy, S. (2018). An evidence-based co-occurring disorder intervention in VA homeless programs: outcomes from a hybrid III trial. *Bmc Health Services Research*, *18*. <https://doi.org/ARTN> 332

10.1186/s12913-018-3123-9

Smith, J. E., Meyers, E. J., & Delaney, H. D. (1998). The community reinforcement approach with homeless alcohol-dependent individuals. *Journal of Consulting and Clinical Psychology*, *66*(3), 541-548. <https://doi.org/Doi> 10.1037/0022-006x.66.3.541

Stahler, G. J., Shipley, T. E., Kirby, K. C., Godboldte, C., Kerwin, M. L. E., Shandler, I., & Simons, L. (2005). Development and initial demonstration of a community-based intervention for homeless, cocaine-using, African-American women. *Journal of Substance Abuse Treatment*, *28*(2), 171-179. <https://doi.org/10.1016/j.jsat.2004.12.003>

Stahler, G. J., Shipley, T. F., Bartelt, D., DuCette, J. P., & Shandler, I. W. (1995). Evaluating alternative treatments for homeless substance-abusing men: Outcomes and predictors of success. *Journal of Addictive Diseases*, *14*(4), 151-167. <https://doi.org/DOI> 10.1300/J069v14n04_09

Theodos, B., Pergamit, M., Derian, A., Edelstein, S., & Stolte, A. (2016). *Solutions for youth: An evaluation of the latin american youth center's promotor pathway program.*

van Kranenburg, G. D., van den Brink, R. H. S., Mulder, W. G., Diekman, W. J., Pijnenborg, G. H. M., & Mulder, C. L. (2019). Clinical effects and treatment outcomes of long-term compulsory in-patient treatment of treatment-resistant patients with severe mental illness and substance-use disorder. *Bmc Psychiatry*, *19*(1). <https://doi.org/ARTN> 270

10.1186/s12888-019-2254-9

Wong, C., Kolodner, K., Fingerhood, M., Bigelow, G. E., & Silverman, K. (2005). *therapeutic workplace for homeless alcohol dependent individuals*.

Wu, Q., Zhang, J., Walsh, L., & Slesnick, N. (2022). Illicit drug use, cognitive distortions, and suicidal ideation among homeless youth: results from a randomized controlled trial. *Behavior Therapy*, *53*(1), 92-104.

Zhang, J., & Slesnick, N. (2018). Substance Use and Social Stability of Homeless Youth: A Comparison of Three Interventions. *Psychology of Addictive Behaviors*, *32*(8), 873-884. <https://doi.org/10.1037/adb0000424>

Zhang, S. X., Shoptaw, S., Reback, C. J., Yadav, K., & Nyamathi, A. M. (2018). Cost-effective way to reduce stimulant-abuse among gay/bisexual men and transgender women: a randomized clinical trial with a cost comparison. *Public Health*, *154*, 151-160. <https://doi.org/10.1016/j.puhe.2017.10.024>
