# Supplementary material for: The effectiveness of psychosocial interventions for reducing problematic substance use, mental ill health, and housing instability in people experiencing homelessness in high income countries: A systematic review and meta‐analysis
Source: Campbell Syst Rev. 2025 Jan 17;21(1):e70019. doi: 10.1002/cl2.70019 (PMC11739802; doi:10.1002/cl2.70019)
Supplement: Supplementary file 3 — Supporting information. [file CL2-21-e70019-s001.docx]

**Appendix – description of relevant psychosocial interventions**

| **Intervention** | **Description** |
| --- | --- |
| 12 Step Facilitation Group | This is an approach intended to increase the chance of someone participating/maintaining participation in a 12 step programme. |
| 12 Step Programmes | These are group based mutual aid interventions aimed at peer support for positive behaviour change and to achieve and maintain abstinence. |
| Brief Interventions | A short term technique used to encourage people to change risky or unhealthy behaviours, including problematic substance use. |
| Brief Motivational Intervention | An intervention which seeks to strengthen a person’s motivation to change risky or unhealthy behaviour. It is a form of Motivational Interviewing. |
| Cognitive Behavioural Therapy | A talking therapy that aims to change how an individual feels, thinks, and behaves, so that the individual can better deal with their problems. |
| Community Reinforcement Approach | This is based on Contingency Management, and draws on environment and community as a way of reinforcing positive behaviours. |
| Contingency Management | A behavioural therapy that uses rewards and punishments to encourage positive behavioural changes. |
| Cue Exposure Treatment | A behavioural intervention in which individuals are exposed to cues to change their conditioned responses. |
| Dialectical Behaviour Therapy | A talking therapy, based on Cognitive Behavioural Therapy, and intended for individuals who feel emotions intensely. It focuses on helping individuals to change while accepting who they are. |
| Family Therapy/Couples Therapy/Community Reinforcement Approach | A behavioural therapy, based on Contingency Management, that draws on family members and partners to reinforce positive behaviours. |
| Mentalisation-Based Therapy | This is a long term talking therapy intervention that aims to help people understand and change how their thoughts and thinking link to behaviours. |
| Motivational Enhancement Therapy | This is a short intervention, based on Motivational Interviewing, and is intended to generate internal motivation is change behaviour. |
| Motivational Interviewing | Is a counselling approach that uses goal-oriented, directional therapy to encourage behavioural change. |
| Non-contingent rewards | A behavioural intervention that provide rewards at regular intervals, and not are dependent on changes in behaviour. |
| Psychodynamic Therapy | This type of therapy aims to help individuals be aware of the unconscious processes that drive their behaviours. |
| Relapse Prevention | This is based on Cognitive Behavioural Therapy, and aims to provide skills to enable individuals to identify and manage situations that increase their chance of relapse. |
| Skills Training | These teach specific skills to help individuals in challenging situations, set goals, understand their thoughts, and change behaviours and use problem solving approaches. |
| SMART | These are group based mutual aid interventions These are group based mutual aid interventions aimed at peer support for positive behaviour change, based on harm reduction approaches (that is, do not require abstinence). |
| Social Behaviour and Network Therapy | A talking therapy that assumes that an individual’s social networks play an important part in encouraging positive behavioural change, but can also influence problematic behaviours. |
| Therapeutic Communities/Residential Rehabilitation | Involve a group of peers with shared goals, working together to enable positive changes in behaviour. |
